# A cell-active cyclic peptide targeting the Nrf2/Keap1 protein–protein interaction†

**DOI:** 10.1039/d3sc04083f

**Published:** 2023-09-20

**Authors:** Jessica Iegre, Sona Krajcovicova, Anders Gunnarsson, Lisa Wissler, Helena Käck, Anna Luchniak, Stefan Tångefjord, Frank Narjes, David R. Spring

**Affiliations:** a Yusuf Hamied Department of Chemistry Lensfield Road CB2 1EW Cambridge UK spring@ch.cam.ac.uk; b Department of Organic Chemistry, Palacky University Olomouc Tr. 17. Listopadu 12 77900 Olomouc Czech Republic; c Mechanistic and Structural Biology, Discovery Sciences, BioPharmaceuticals R&D, AstraZeneca Pepparedsleden 1 43183 Gothenburg Sweden; d BioScience, Research & Early Development, Respiratory & Immunology, BioPharmaceuticals R&D, AstraZeneca Pepparedsleden 1 43183 Gothenburg Sweden; e Medicinal Chemistry, Research & Early Development, Respiratory & Immunology, BioPharmaceuticals R&D, AstraZeneca Pepparedsleden 1 43183 Gothenburg Sweden frank.narjes@astrazeneca.com

## Abstract

The disruption of the protein–protein interaction (PPI) between Nrf2 and Keap1 is an attractive strategy to counteract the oxidative stress that characterises a variety of severe diseases. Peptides represent a complementary approach to small molecules for the inhibition of this therapeutically important PPI. However, due to their polar nature and the negative net charge required for binding to Keap1, the peptides reported to date exhibit either mid-micromolar activity or are inactive in cells. Herein, we present a two-component peptide stapling strategy to rapidly access a variety of constrained and functionalised peptides that target the Nrf2/Keap1 PPI. The most promising peptide, P8-H containing a fatty acid tag, binds to Keap1 with nanomolar affinity and is effective at inducing transcription of ARE genes in a human lung epithelial cell line at sub-micromolar concentration. Furthermore, crystallography of the peptide in complex with Keap1 yielded a high resolution X-ray structure, adding to the toolbox of structures available to develop cell-permeable peptidomimetic inhibitors.

## Abstract 2

Application of DVP stapling methodology to efficiently develop Nrf2 peptides with nM activity in enzymatic assays, submicromolar activity in cells and non-cytotoxicity, characterised by high resolution X-ray co-crystallography.

## Graphical abstract

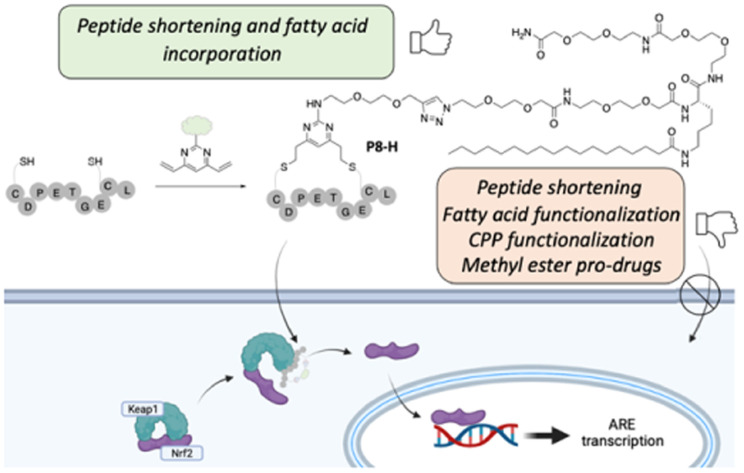

## Introduction

**Fig. 1:**
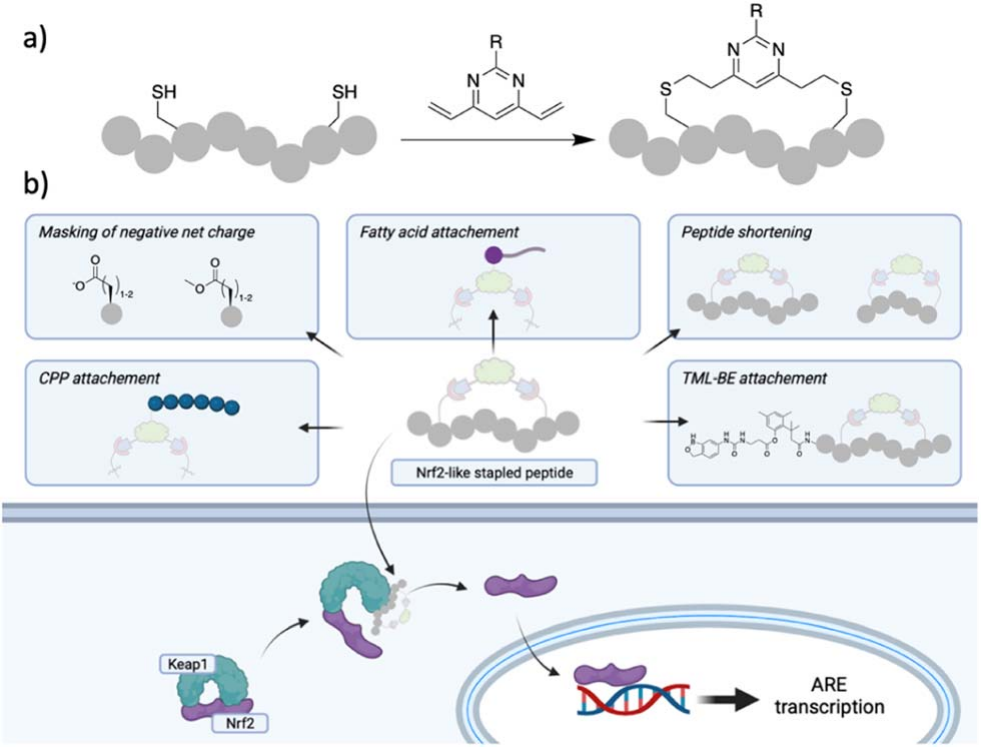
(a) DVP peptide stapling methodology successfully used in this work; (b) strategies used in this work to aid cell permeability of Nrf-2 like peptides following 2C-PS with DVP staples.

**Fig. 2:**
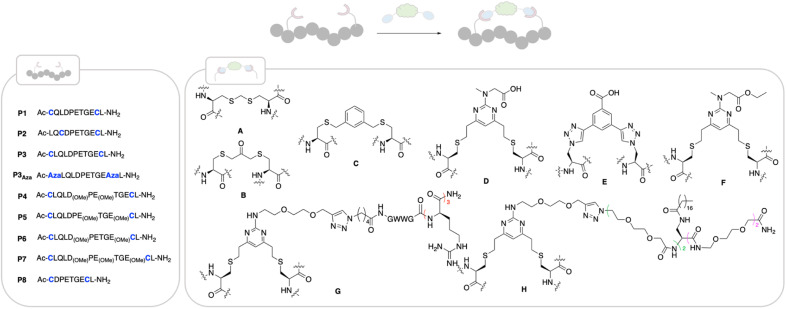
Structures of peptides made in this work. Stapling positions on the different peptides are coloured in blue. Peptides were made using SPPS (ESI†) and cyclised as follows: cyclisation with staple A: linear peptide (1 eq.), H_2_O, TCEP·HCl (1.5 eq.), K_2_CO_3_ (3 eq.) then NEt_3_ (10 eq.), diiodomethane (8 eq.), THF, rt, 4 h; cyclisation with staple B: linear peptide (1 eq.), 50 mM NaPi buffer pH 8, TCEP·HCl (1.5 eq.), dichloroacetone (3 eq.), DMF, rt, 4 h,; cyclisation with staple C: linear peptide (1 eq.), TCEP·HCl (1.5 eq.), 50 mM NaPi pH 8, rt, 1 h, then 1,3-bisdibromomethylbenzene (3 eq.), DMF, rt, 4 h; cyclisation with DVP staples D–H: linear peptide (1 eq.), 50 mM NaPi buffer, pH 8, 5% DMF, staple D–H (1.1 eq.), rt, 1 h.

The transcription factor Nuclear Factor Erythroid-2-related factor (Nrf2) protects the cells against oxidative stress by regulating the transcription of more than 200 genes containing the antioxidant response element (ARE). Nrf2 is negatively regulated by the protein–protein interaction (PPI) with the E3 ligase Kelch-like EH-associated protein 1 (Keap1), which as part of a Cullin3-RBX1 complex binds Nrf2 in the cytosol, promoting its ubiquitination and proteasomal degradation.^1^ Oxidative stress is recognised as a major driver in a variety of pathological conditions including cancer, neurodegenerative conditions, chronic obstructive pulmonary disease (COPD) and other inflammatory-driven diseases.^2^ Disruption of the Nrf2/Keap1 PPI is regarded as a promising strategy to upregulate Nrf2 levels and protect cells against oxidative stress and small molecule as well as peptide approaches have been pursued to achieve this goal.^3,4^ Nrf2 interacts with Keap1 *via* two conserved motifs in the Nrf2-ECH homology domain 2 (Neh2), namely DLG and ETGE. Unlike the DLG, the ETGE motif binds strongly to Keap1 (KD ∼ 5 nM) through H-bonds, electrostatic and van der Waals interactions.^5,6^ Of particular interest for targeting this PPI are peptide inhibitors since they resemble the structure of the epitope of Nrf2 interacting with Keap1. The hydrophilic binding site on Keap1 requires charged and polar sequences for high affinity interactions with Nrf2, making cell permeability an issue. A plethora of potent linear or cyclic peptides, with or without moieties to enhance cell permeability have been reported thus far; however, they either lack or show a significant drop-off in activity in cellular assays.^7–23^ The limited cellular activity hampers the use of Nrf2-like peptides as effective Keap1/Nrf2 inhibitors. On the other hand, small molecule Nrf2/Keap1 inhibitors can overcome the cell permeability issues.^24^ However, due to the plasticity of the Keap1 binding site, they can target different conformations of the Keap1 protein, and represent a complementary approach to Keap1/Nrf2 inhibition.^25–30^ Consequently, the development of a cell permeable Nrf2-like peptide that retains activity in the cellular environment would be a significant breakthrough. Herein, we describe a variety of Cys dependant stapling approaches of an Nrf2 peptide, of which the divinylpyrimidine (DVP)^31^ two-component peptide stapling (2C-PS) approach proved particularly useful to easily access a variety of peptides of different sizes carrying different functionalities. The most promising of the initial peptides, P3-F, was further optimised into a cell permeable peptide. To this end, we adopted different strategies and combinations thereof, namely peptide shortening, replacement of acidic residues with methyl esters, poly-arginine residues with the endosomal escape enhancer motif GWWG and incorporation of fatty acid tags (Fig. 1).^32–35^ Peptide P8-H (Fig. 2; shorter peptide containing a stearic tag) retained its binding affinity in the enzymatic assays alongside exhibiting a submicromolar EC_50_ value (0.75 μM) in a cellular assay. Peptide P8-H represents, to the best of our knowledge, the most potent Nrf2/Keap1 peptide inhibitor with cellular activity known to date. Moreover, we were able to obtain X-ray structures of Keap1 in complex with peptide P3-F and the shortened analogue P8H. The binding mode was found to maintain the hotspot interactions of the ETFG motif of the native epitope. These could present useful starting points for the design of peptidomimetics.^36,37^

## Results and discussion

We based the design of our Nrf2-like cyclic peptides around the amino acid sequence of the ETGE motif (namely 12-mer ^73^QLQLDEETGEF^84^L) and SAR studies reported in the literature.^9,10,12,14^ The ETFE motif is known to adopt a beta-turn conformation upon binding to Keap1. Residues Q73-Q75 and L85 stabilise the bound conformation, L76-D77 and E79-G81 bind deep into the pocket with E79 and E82 forming electrostatic interactions with Keap1 and D77 and T80 stabilising the beta-turn *via* H-bonds with E78 not contributing to the binding. In particular, our peptides aim to retain the beta-turn conformation and featured the E78P mutation which has been reported to improve the binding affinity.^9^ Visual inspection of the X-ray structures of previously published peptides in complex with Keap1 and of the native Keap1/Nrf2 PPI (PDB: 4IFL,^10^5WFV,^38^2FLU^6^) suggested a combination of three sequences, P1–P3 and five different staples (A–E) to initiate our investigation (Fig. 2). In all three sequences, F83 was replaced with Cys or azido alanine (Aza), since it is well-established that this residue does not contribute to binding affinity.^38^ The ability of the cyclic peptides to bind to the Kelch domain of Keap1 was evaluated using surface plasma resonance (SPR), where the Kelch domain is immobilised onto a SPR sensor chip. Additionally, we assessed the ability of the peptides to inhibit the PPI between Nrf2 and the Kelch domain of Keap1 using a time-resolved FRET (TR-FRET) assay. Here, the IC_50_ for inhibition of the interaction between the human Keap1 Kelch domain and an Nrf2-ETGE peptide is measured.

Compared to the 11-mer ^74^LQLDEETGEF^84^L peptide (entry 1, Table 1), which had a *K*_D_ value of 52 nM and inhibited the TR-FRET assay with an IC_50_ of 5.3 nM, P1 and the P3 derived cycles showed approximately 2–3-fold improvement in binding affinity, nearly irrespective of the staple used.

**Table 1:** SPR and TR-FRET results of the Nrf2-like peptides. *K*_D_ and IC_50_ values are the geometric mean ± sd of at least 3 independent repeats, unless otherwise stated; n.d. = not determined. Peptides P1, P2, P3 oxidised over time as detected by LCMS^c^

Entry	Sequence	SPR *K*_d_ (nM)	SPR *k*_on_ (M^−1^ s^−1^) *k*_off_ (s^−1^)	TR-FRET IC_50_ (nM)
1	^74^LQLDPETGEF^83^L	52 ± 1.4	1.7 × 10^5^/7.9 × 10^−3^	5.3 ± 1.2
2	P1	20 ± 1.2^a^	8.6 × 10^4^/1.4 × 10^−3^	4.9 ± 1.1
3	P1-A	19 ± 1.4	4.8 × 10^5^/7.3 × 10^−3^	2.9 ± 1.7
4	P1-B	27 ± 1.3	4.1 × 10^5^/8.7 × 10^−3^	4.4 ± 1.4
5	P1-C	17 ± 1.2^a^	4.7 × 10^5^/7.8 × 10^−3^	2.1^b^
6	P1-D	25 ± 1.3	2.0 × 10^5^/4.7 × 10^−3^	2.9 ± 1.4
7	P2	75	n.d.^c^	n.d.
8	P2-A	70 ± 1.0	2.1 × 10^5^/1.5 × 10^−2^	8.3 ± 1.1
9	P2-B	97 ± 1.4	3.5 × 10^5^/2.8 × 10^−2^	9.8 ± 1.2
10	P2-C	36 ± 1.8^a^	5.0 × 10^5^/1.3 × 10^−2^	5.6 ± 1.1
11	P2-D	54 ± 1.2	1.6 × 10^5^/8.3 × 10^−3^	4.8 ± 1.5
12	P3	19 ± 1.8	1.8 × 10^5^/4.9 × 10^−3^	5.0 ± 1.5
13	P3Aza-E	34 ± 1.3	1.7 × 10^5^/5.4 × 10^−3^	4.0 ± 1.4
14	P3-D	22 ± 1.3	1.4 × 10^5^/2.8 × 10^−3^	2.4 ± 1.3
15	P3-F	14 ± 1.4	2.4 × 10^5^/3.2 × 10^−3^	1.9 ± 1.3

a Tested twice.

b Tested once.

c Linear peptides P1, P2 and P3 containing free cysteines, were synthesised as starting material for the cyclised peptides. Their purity declined overtime, presumably due to intra- or intermolecular disulphide formation. The data for P1 and P3 were comparable over several testing occasions, whereas for *b* we were not able to obtain reproducible *K*_D_'s or IC_50_'s.

Cyclisation thus did not improve the affinity to a significant extent compared to the linear peptide, as previously reported. One reason for this is the preorganisation of the linear Keap1 peptide into a β-turn, which is even more pronounced by the presence of P78.^39^ On- and off rates were comparable across the two series. Pleasingly, in the TR-FRET assay, the P1 and P3 cyclic peptides showed nM enzymatic inhibition with IC_50_ ranging from 2 to 5 nM (Table 1). Conversely, the analogues in P2 series, having a smaller cycle size, had equal or less affinity compared to the parent peptide, which might be due to a faster off-rate for several analogues, when compared to the matched pairs in the P1 and P3 series. Among all the cyclic unmodified peptides, P3-D (*K*_D_ = 22 nM and IC_50_ = 2.4 nM) was selected for further investigation due to the functional handle on its staple, that allows for facile elaboration.

To avoid the additional negative charge brought by the carboxylic acid on staple D, we changed the residue to an ethyl ester to yield P3-F (Fig. 2), which displayed similar activity displaying a *K*_D_ of 12 ± 1 nM and an enzymatic IC_50_ of 2 ± 1.3 nM (Table 1).

**Fig. 3:**
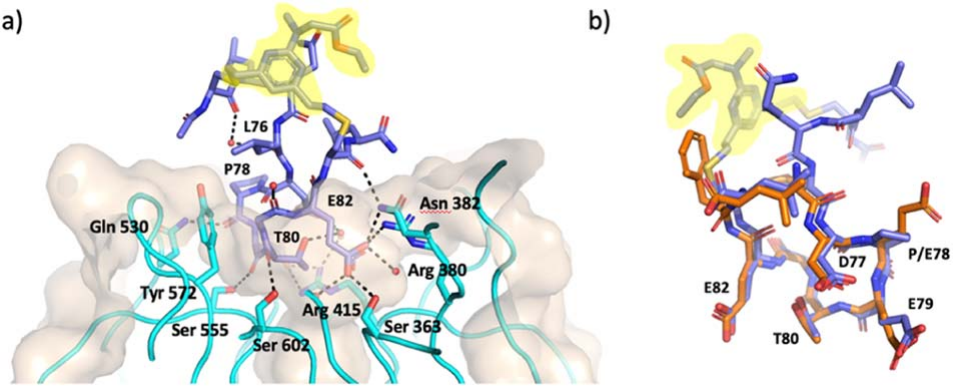
X-ray crystal structure of P3-F bound to Keap1 (PDB 8Q1Q, 1.4 Å). (a) Structure of P3-F (purple) in complex with Keap1 (cyan ribbons and beige surface) key interactions of P3-F hotspot with water molecules and Keap1 residues in the binding site are also shown. (b) Overlay of P3-F (purple) with the native peptide (orange, PDB: 5WFV). Staple highlighted in yellow.

An X-ray crystal structure of P3-F bound to Keap1 Kelch domain could be obtained and allowed to investigate its binding mode (Fig. 3). The peptide was found to bind to the Kelch domain of Keap1 and maintained the conformation of the backbone and the side-chains when compared to the structure of linear peptides (PDB: 5WFV and 2FLU).^6,38^ This includes the key interactions made by E79 with residues R415, R483 and S508 in Keap1 and of E82 with R380, N382 and S363, as well as the intramolecular hydrogen bonds established by T80 and D77 to stabilise the turn structure.^6,38^ The staple and the flanking residues are protruding from the binding site, lacking direct interactions with Keap1 and are consequently less ordered, as demonstrated by less defined electron density for this portion of P3-F (Fig. 3).

The effect of peptides on Nrf2-dependent gene expression was investigated in BEAS-2B cells, where we determined the upregulation of the mRNA of NAD(P)H quinone dehydrogenase (NQO1) by qPCR. The expression of NQO1 is Nrf2-dependent and it is one of the two major quinone reductases, which play a crucial role in protecting cells from oxidative stress.^40^§§In the context of this study we chose BEAS-2B cells as a representative model for epithelial cells. The presence of serum has been reported to affect BEAS-2B cell phenotype. Specifically, exposure of BEAS-2B to FBS has been associated with squamous differentiation, alteration in cytokine secretion and response to toxicants.^42–44^ Therefore, we did not use FBS in the cell assay media. Incubation of peptides P1-A, P1-D, P2-A, P2-D, P3-D, P3-F as well as the linear 11-mer peptide for 24 h did not lead to any induction of NQO1 mRNA at concentrations of up to 10 μM, whereas a small molecule inhibitor of the Keap1–Nrf2 interaction had an EC_50_ = 12 nM, matching the reported value.^25^ The lack of activity of the peptides is unsurprising considering the overall negative net charge, which will obstruct their cellular uptake. In order to improve the cellular uptake, we took of advantage of the 2C-PS moiety, which not only allows the easy synthesis of a variety of macrocycles of different ring sizes, but also permits the facile introduction of a diverse set of moieties, which could enhance cell permeability. We pursued a diverse set of strategies to make the Keap1 peptide more cell permeable, namely: masking of the acidic amino acids with methyl esters, introduction of polyarginine peptides, GWWG-polyarginine peptides or a long-chain fatty acid tag onto the staple, as well as peptide shortening and a combination of these. The rationale behind these strategies is explained herein, and details of their synthesis can be found in the ESI.†

### Methyl esters to mask the acidic residues

It was hypothesised that by masking the Glu and Asp residues as methyl esters would allow peptides to overcome hydrophobic barriers more easily and hence facilitate cell penetration. Consequently, we synthesised four ester bearing peptides P4-F, P5-F, P6-F and P7-F, where different combinations of the carboxylates were masked as methyl esters. The Glu and Asp residues are deemed as critical to the binding, and hence, it was not surprising to see that the ester-bearing peptides showed reduced activity in both enzymatic assays (Table 2, entries 1 and 4). It was thought that upon cellular internalisation, intracellular carboxyl esterase would hydrolyse the methyl esters to the free carboxylic acids,^41^ leading to inhibition of the Keap1–Nrf2 interaction and expression of NQO1.

**Table 2:** SPR, HTRF and BEAS-2B results of the functionalised peptides. *K*_D_ values are the geometric mean ± sd of at least two independent repeats, for TR-FRET a minimum of three repeats was obtained, unless stated otherwise^c^

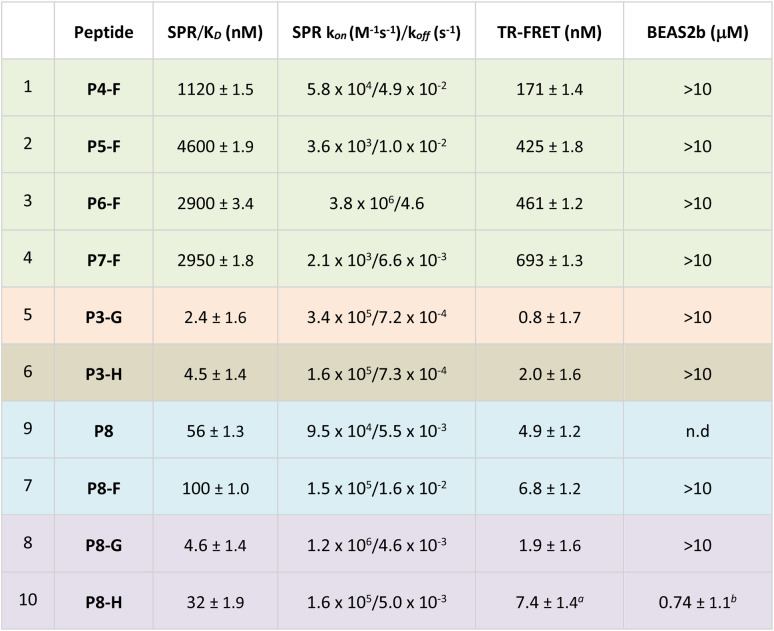

a *n* = 8.

b *n* = 5.

c Green = carboxylic acid masking strategy; orange = CPP tag strategy; brown = fatty acid tag strategy; blue = peptide shortening strategy; purple = combination of several strategies.

Disappointingly, no Nrf2-dependent gene expression was observed in the cell assay, indicating either lack of internalisation or lack of intracellular methyl ester hydrolysis.

### Incorporation of GWWG-polyarginine peptide onto the staple

Another strategy to enhance the cell permeability of peptides is the addition of a tri-(L)Arg motif onto the staple coupled with GWWG sequence. Tri-(L)Arg tags were previously reported to be able to enhance the cellular uptake of stapled peptides into cells, while the GWWG motif is a hydrophobic sequence known to enhance the endosomal escape.^33,34^ Therefore, synthesis of peptides P3-G and P7-G was attempted (Fig. 2). Solubility issues hampered the synthesis of P7-G whereas peptide P3-G was successfully synthesised. Despite showing low nM activity in the biophysics and enzymatic assays (Table 2), no activity was detected in the cellular experiments.

### Peptide shortening

**Fig. 4:**
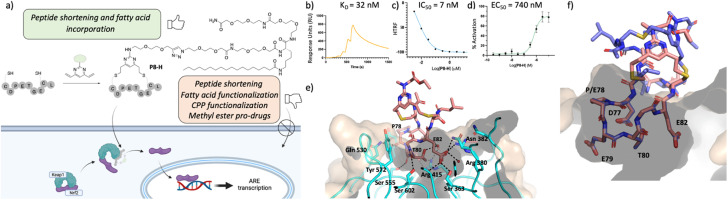
Profiling of P8-H. (a) structure of the lead peptide obtained vie DVP peptide stapling and it's proposed mechanism of action; (b) SPR data of the peptide showing a *K*_D_ of 92 nM in two independent repeats; (c) HRFT data of the peptide shown as a geometric mean of 3 independent repeats; (d) qPCR results in BEAS-2B cells shown as the SEM of 5 independent repeats; (e) crystal structure of peptide P8-F (pink, PDB: 8Q1R) in complex with Keap1. (f) Overlay of P8-F (pink) with P3-F (purple). While P8-F is shorter, as designed, all the key interactions with Keap1 are maintained.

Guided by the X-ray structure of P3-F in complex with Keap1, we envisaged that the removal of amino acids not essential for Keap1 binding would result in reduction of the number of hydrogen-bond donors that might limit membrane permeability. Therefore, it was decided to make use of the shorter sequence obtainable by removing the two N-terminal residues of P2-D, leading to P8 and sequentially cyclise the peptide using staples F and G (Fig. 2 and 4). Peptide P8-F showed a *K*_D_ of 100 nM in the SPR assay, in line with the values seen for the P2 analogues. We believe the higher affinity observed for P8-G to be due to interaction of the tag with the SPR surface. Both peptides showed low nM inhibition in the TR-FRET assay.

Pleasingly, an X-ray structure of P8-F revealed identical binding of the ETGE motif to Keap1 as compared with P3-F, confirming the design hypothesis (Fig. 4e and f). Different from the larger P3-F macrocycle, the staple in P8-F is in closer proximity to Keap1, and a hydrogen bond between the thioether and the phenol oxygen of Tyr334 of 3.4 Å can be observed (Fig. SI_1†). However, shortening of the peptide on its own (P8-F) or combining peptide shortening with the cell penetrant staple G (P8-G) did not lead to any activity in the qPCR assay. Shortening of the methyl ester containing peptides was not possible due to solubility issues encountered during their synthesis.

### Incorporation of C18 motif onto the staple

We decided to modify the staple by incorporating a C18 motif (H, Fig. 2 and 4a). Fatty acid tags have been reported to effectively transport small molecules and peptides into cells,^35^ and therefore, this was regarded as a reasonable strategy to transport the Nrf2 peptides into cells (Fig. 4). The C18 motif to attach to the stapled peptides P3-H and P8-H was designed in such a way that it could be easily made on by solid phase peptide synthesis and would overcome solubility issues, often encountered in the synthesis of the C18-bearing stapled peptides.¶¶Synthesis of the ester bearing peptides and corresponding shorter analogues was not possible due to solubility issues.P3-H and P8-H showed nM activity in the SPR and HTRF assays with *K*_D_'s of 4 and 32 nM and IC_50_'s of 2 and 7 nM respectively. Comparable *k*_ON_ and *k*_OFF_ rates were observed for both peptides (Table 2). Whilst the more potent, but larger macrocycle did not induce Nrf2-dependent gene expression, we were pleased to observe that the shorter peptide P8-H, was active in BEAS-2B cells with an EC_50_ of 740 nM. In addition, P8-H showed no cytotoxicity when tested in a CellTiter Glo assay up to 30 μM (Fig. SI_2†). These results suggest that the peptide disrupts the Nrf2/Keap1 PPI and acts as a chemical probe since it engages Keap1 in both the enzymatic and cellular settings and structural information is available.

## Conclusions

In this work, we showcased the potential of the DVP-2C peptide stapling to access a variety of functionalised Nrf2-like peptides. Peptide P3-F, featuring a simple functionalisation on the staple, yielded an X-ray structure in complex with Keap1 of a high resolution (1.4 Å). The structure revealed that the peptide retains the hotspot of the native epitope whilst the staple and functionality sit outside the binding site. Furthermore, the high resolution allowed us to accurately observe the position of each atom and water network, serving as an excellent starting point for the design of peptidomimetic inhibitors. The DVP-2C stapling methodology was also well-suited in the further functionalisation of the cyclic peptides. Among the strategies explored to aid cell penetration, the combination of peptide shortening and fatty acid tag was the most promising. In addition to exhibiting low nM activity in the enzymatic assays, stapled peptide P8-H, functionalised with a stearic fatty acid, was able to induce the expression of the Nrf2-dependent gene NQO1 in cells at submicromolar concentration – the most promising cellular activity reported to date for a peptide. The ease of functionalisation and exploration of different macrocycle sizes coupled with the high-resolution X-ray structure and the submicromolar cellular activity make the peptides presented in this work an excellent starting point to develop further peptide Nrf2/Keap1 inhibitors.

## Data availability

The coordinates and structure factors have been deposited to the Protein Data Bank (PDB) with accession codes, 8Q1Q and 8Q1R.

## Author contributions

JI: project conception, performed experiments, wrote the manuscript; SK: performed experiments, approved the manuscript; AG: performed experiments, approved the manuscript Lisa Wissler – performed experiments, approved the manuscript; HK: performed experiments, approved the manuscript; AL: performed experiments, approved the manuscript; ST: performed experiments, approved the manuscript; FN: project conception, project supervision, wrote the manuscript; DRS: project conception, project supervision, approved the manuscript.

## Conflicts of interest

A. G., L. W., H. K., A. L., S. T. and F. N. are AstraZeneca full-time employees and may own stock or stock options.

## Supplementary Material

SC-014-D3SC04083F-s001
