# Supplementary material for: A cell-active cyclic peptide targeting the Nrf2/Keap1 protein–protein interaction
Source: Chem Sci. 2023 Sep 20;14(39):10800–5. doi: 10.1039/d3sc04083f (PMC10566475; doi:10.1039/d3sc04083f)
Supplement: SC-014-D3SC04083F-s001 [file SC-014-D3SC04083F-s001.pdf]

Electronic Supplementary Material (ESI) for Chemical Science.
This journal is © The Royal Society of Chemistry 2023

Supporting information

A cell-active cyclic peptide targeting the Nrf2/Keap1 protein-protein interaction

Chemistry experimental 2

- Small molecules synthesis and characterization 4
- NMR spectra 8
- Peptide synthesis 13
- Peptide characterization 18

Biophysics experimental 21

- Protein Production 21
- Surface Plasmon Resonance 21
- HTRF 21
- X-ray crystallography 22

Cellular biology experimental 25

- Nrf2 dependent gene expression qPCR 25
- CellTiter Glo assay 25

Chemistry experimental

All experiments were carried using distilled solvents unless otherwise stated.

Reagents: Chemicals were purchased from commercial sources and used without further purification.

Yield: refer to chromatographically and spectroscopically pure compounds unless otherwise stated and are reported as follows: mass, moles, percentage.

Temperature: Reaction temperatures of 0 °C were maintained using an ice-water bath; room temperature (rt) refers to 20-25 °C.

Flash chromatography: Analytical thin layer chromatography was carried out on SiO_2 Merck Kieselgel 60 F254 plates with visualisation either by ultraviolet light or staining with potassium permanganate or ninhydrin dips made using standard procedures. Retention factors (R_f) are quoted to 0.01. Flash column chromatography was performed using silica gel 60 (230-400 mesh), under a positive pressure. Eluent systems are expressed in % v/v.

Nuclear Magnetic Resonance (NMR): ^{1}H, ^{13}C and spectra were recorded using an internal deuterium lock at ambient probe temperatures on the following instruments: Bruker Avance III 400 MHz HD Smart Probe Spectrometer, Bruker Avance III 400 MHz HD Spectrometer, Bruker 400 MHz QNP Cryoprobe Spectrometer, Bruker 500 MHz DCH Cryoprobe Spectrometer, Bruker Avance III 500 MHz HD Smart Probe Spectrometer. The following deuterated solvents were used: chloroform ($CDCl_3$), dimethylsulfoxide (DMSO-d_6) and methanol (CD_3OD). ^{1}H NMR chemical shifts (δ) are quoted in ppm to the nearest 0.01 ppm, relative to the residual non-deuterated solvent peak and coupling constants (J) are quoted to the nearest 0.1 Hertz (Hz). ^{13}C NMR chemical shifts are quoted to the nearest 0.1 ppm, relative to the solvent peak and coupling constants are quoted to the nearest 0.1 Hz. ^{19}F NMR chemical shifts are quoted to the nearest 0.1 ppm. Spectral data is reported as follows: chemical shift, multiplicity (s, singlet; d, doublet; dd, doublet of doublets; t, triplet; q, quartet; sept, septet; m, multiplet; br, broad; or as a combination of these *e.g.* br s, dd, dt), coupling constant(s) and integration.

Infra-red spectroscopy (IR): Infra-red spectra were recorded neat on a Perkin Elmer Spectrum One FT-IR spectrometer fitted with an Attenuated Total Reflectance (ATR) sampling accessory. Selected absorption maxima (ν_{max}) are quoted in wavenumbers (cm^{-1}) with the following abbreviations: w, weak; m, medium; s, strong; vs, br, broad.

Liquid chromatography-mass spectrometry (LCMS): LCMS was carried out using a Waters ACQUITY H-Class UPLC with an ESCi Multi-Mode Ionisation Waters SQ Detector 2 spectrometer using MassLynx

4.1 software; ESI refers to the electrospray ionisation technique; LC system: solvent A: 2 mM NH_4OAc in H_2O/MeCN (95:5); solvent B: MeCN; solvent C: 2% formic acid; column: ACQUITY UPLC® CSH C18 (2.1 mm × 50 mm, 1.7 µm, 130 Å) at 40 °C; gradient: 5 – 95% B with constant 5% C over 1 min at flow rate of 0.6 mL/min; Injection volume: 5 µL. Chromatographs were monitored by absorbance using diode array detection at a wavelength range of 190-600 nm, interval 1.2 nm.

High resolution mass spectrometry (HRMS): HRMS was carried out using a Waters LCT Premier® Time of Flight (ToF) mass spectrometer or the ThermoFinnigan Orbitrap Classic mass spectrometer. Reported mass values are within the error limits of ± 5 ppm mass units. ESI refers to the electrospray ionisation technique.

Analytical HPLC: Chromatographs were obtained on an Agilent 1260 Infinity® using a reversed-phase Supelcosil ABZ+PLUS column (150 mm x 4.6 mm, 3 µm) eluting with a linear gradient system (solvent A: 0.05% (v/v) TFA in water, solvent B: 0.05% (v/v) TFA in MeCN) over 15 min, unless otherwise stated, at a flow rate of 1 mL/min. HPLC was monitored by UV absorbance at 220 and 254 nm.

Preparative HPLC: Preparative HPLC was carried out on an Agilent 1260 Infinity® using a reversed-phase Supelcosil ABZ+PLUS column (250 mm x 21.2 mm, 5 µm) eluting with a linear gradient system (solvent A: 0.1% (v/v) TFA in water, solvent B: 0.05% (v/v) TFA in MeCN) over 20 min at a flow rate of 20 mL/min. HPLC was monitored by UV absorbance at 220 and 254 nm.

Small molecules synthesis and characterization

Staples **D**, **F** and **5** were prepared according to the generic synthetic route illustrated below (Scheme S1)

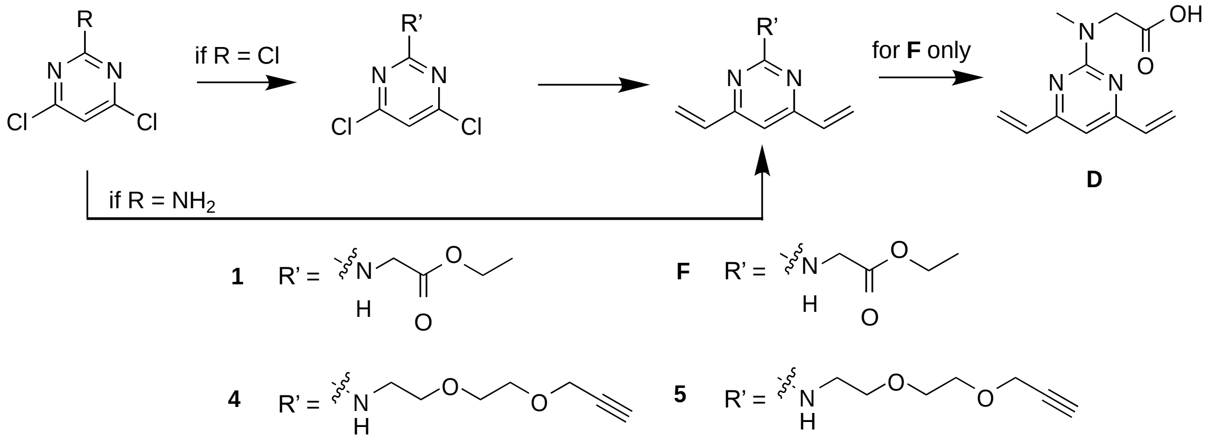

*Scheme S1 - Generic synthetic routes to the staples presented in this work. Staple **5** was stapled to the peptides of interest before being further functionalised to staples **G** and **H** as shown in Scheme ESI_2*

Ethyl *N*-(4,6-dichloropyrimidin-2-yl)-*N*-methylglycinate (1)

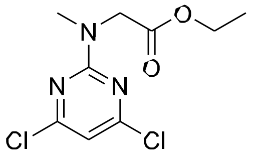

To a solution of 2,4,6-Trichloropyrimidine (1.00 g, 5.45 mmol) in acetone (6 mL) at 0° C was added sarcosine ethyl hydrochloride (1.01 g, 6.54 mmol) followed by dropwise addition of triethylamine (1.90 mL, 13.6 mmol) and the reaction mixture was stirred at 0° C for 2 h and then at rt for 12 h. Upon completion, the solvent was removed *in vacuo* then redissolved in H_2O (20 mL) and extracted with CH_2Cl_2 (4 × 20 mL). The combined organic fractions were dried ($MgSO_4$), concentrated *in vacuo* and the crude residue purified by FCC (2-20% EtOAc/PE) to yield the product (200 mg, 0.80 mmol, 14%) as a clear oil.

R_f 0.30 (10% EtOAc/PE 40-60); **δ_H** (400 MHz, $CDCl_3$) 6.61 (s, 1H), 4.35 (s, 2H), 4.23 (q, 2H, *J* = 7.2 Hz), 3.58 (s, 3H), 1.30 (t, 3H, *J* = 7.2 Hz) ppm; **HRMS** (ESI) *m/z* found $[M+H]^+$ 264.0299, $C_9H_{12}Cl_2N_3O_2^+$ required 264.0307.

Data in accordance with literature procedure.[1]

Ethyl *N*-(4,6-divinylpyrimidin-2-yl)-*N*-methylglycinate (F)

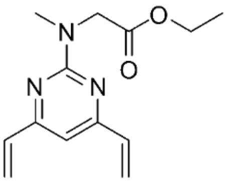

1 (180 mg, 0.638 mmol), Potassium vinyltrifluoroborate (581 mg, 4.34 mmol), Pd(dppf)Cl_2 ▪ CH_2Cl_2 (73 mg, 0.10 mmol) and K_2CO_3 (660 mg, 4.08 mmol) in THF/H2O (10:1, 4.8 mL) were refluxed at 70 °C for 16 h. Upon completion, the reaction mixture was filtered through Celite®, washed with EtOAc and the solvent removed *in vacuo*. The resulting residue was purified by FCC (0-4% EtOAc/PE 40-60) to yield **F** (150 mg, 0.60 mmol, 95%) as an off-white solid.[2]

R_f 0.30 (10% EtOAc/PE 40-60); **δ_H** (400 MHz, $CDCl_3$) 6.64-6.57 (m, 2H), 6.51 (s, 1H), 6.40 (d, 2H, *J* = 17.2 Hz), 5.55 (dd, 2H, *J* = 10.5, 1.0 Hz), 4.38 (s, 2H),4.19 (q, 2H, *J* = 7.1 Hz), 3.34 (s, 3H), 1.30-1.24 (m, 3H) ppm; **HRMS** (ESI) *m/z* found $[M+H]^+$ 248.1397, $C_{13}H_{18}N_3O_2^+$ required 248.1399.

Data in accordance with literature procedure.[1]

***N*-(4,6-Divinylpyrimidin-2-yl)-*N*-methylglycine (D)**

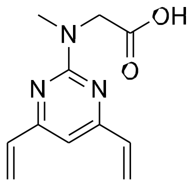

To a solution of **F** (160 mg, 0.65 mmol) in THF/H_2O (1:1, 6 mL) was added LiOH ▪ H_2O (90 mg, 2.14 mmol) and the reaction mixture stirred at rt for 18 h. Upon completion, the mixture was diluted with H_2O (10 mL) and washed with Et_2O (10 mL). The aqueous phase was neutralized with 1M HCl and extracted with CH_2Cl_2 (4 × 20 mL). The combined organic fractions were dried ($MgSO_4$) and concentrated *in vacuo*. The crude residue was triturated with PE 40-60 to yield **3** (100 mg, 0.45 mmol, 70%) as a pale yellow solid.

[1] A general approach for the site-selective modification of native proteins, enabling the generation of stable and functional antibody-drug conjugates, S. J. Walsh, S. Omarjee, W. R. J. D. Galloway, T. T.-L. Kwan, H. F. Sore, J. S. Parker, M. Hyvonen, J. S. Carroll, D. R. Spring, *Chem. Sci.* **2019**, *10*, 694-700

[2] The pure product was dissolved straightaway in THF/H_2O (2 mL) for the next step.

δ_H (400 MHz, $CDCl_3$) 6.67-6.60 (m, 3H), 6.42 (dd, 2H, *J* = 17.3, 0.8 Hz), 5.66 (dd, 2H, *J* = 10.6, 1.2 Hz), 4.32 (s, 2H), 3.40 (s, 3H) ppm; **LCMS** (ESI) 220.1 $[M+H]^+$

Data in accordance with literature procedure.[1]

4,6-Dichloro-*N*-(2-(2-(prop-2-yn-1-yloxy)ethoxy)ethyl)pyrimidin-2-amine (4)

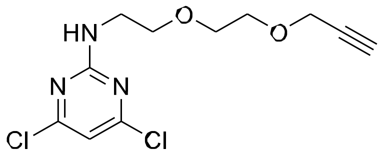

To a solution of 2,4,6-Trichloropyrimidine (1.00 g, 5.45 mmol) in acetone (6 mL) at 0° C was added 2-(2-(prop-2-yn-1-yloxy)ethoxy)ethan-1-amine (0.9 mL, 6.50 mmol) followed by dropwise addition of triethylamine (1.90 mL, 13.6 mmol) and the reaction mixture was stirred at 0° C for 2 h and then at rt for 12 h. Upon completion, the solvent was removed *in vacuo* then redissolved in H_2O (20 mL) and extracted with CH_2Cl_2 (4 × 20 mL). The combined organic fractions were dried ($MgSO_4$), concentrated *in vacuo* and the crude residue purified by FCC (2-50% EtOAc/PE 40-60) to yield the product (292 mg, 1.07 mmol, 20%) as **4** a yellow oil.

R_f 0.27 (10% EtOAc/PE 40-60); **ν_{max}** 3301 (w), 2870 (w), 1530 (str), 1519 (str), 1447 (m), 1346 (w), 1251 (m), 1211 (w), 1097 (str), 1032 (w), 993 (w), 813 (m), 659 (w) cm^{-1}; **δ_H** (400 MHz, $CDCl_3$): 6.61 (s, 1H), 5.89 (br s, 1H), 4.23 (d, 2H, *J* = 2.4 Hz), 3.73-3.68 (m, 4H), 3.67-3.64 (m, 4H), 2.47 (t, 1H, *J* = 2.4 Hz) ppm; **δ_C** (101 MHz, $CDCl_3$): 161.6, 108.9, 79.5, 74.7, 70.3, 69.5, 69.0, 58.5, 41.3 ppm; **HRMS** (ESI) calculated for $[C_{11}H_{14}Cl_2N_3O_2]^+$ 290.0458 *m/z* found 290.0460 $[M+H]^+$

***N*-(2-(2-(Prop-2-yn-1-yloxy)ethoxy)ethyl)-4,6-divinylpyrimidin-2-amine (5)**

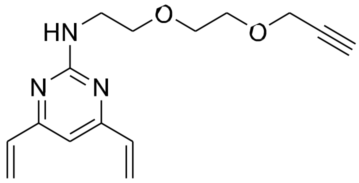

4 (266 mg, 0.92 mmol), potassium vinyltrifluoroborate (616 mg, 4.6 mmol), $Pd(dppf)Cl_2$ ▪ CH_2Cl_2 (67 mg, 0.09 mmol) and Na_2CO_3 (243 mg, 2.3 mmol) in THF (6 mL) were refluxed at 70 °C for 16 h. Upon completion, the reaction mixture was filtered through Celite®, washed with EtOAc and the solvent removed *in vacuo*. The resulting residue was purified by column chromatography (50% EtOAc/CH_2Cl_2) to yield **5** (54 mg, mmol, 22%) as a yellow oil.

R_f 0.15 (10% EtOAc/PE 40-60); **ν_{max}** 3301 (w), 2870 (w), 1530 (str), 1519 (str), 1447 (m), 1346 (w), 1251 (m), 1211 (w), 1097 (str), 1032 (w), 993 (w), 922 (w), 813 (m), 777 (m), 659 (w) cm^{-1}; **δ_H** (400 MHz, $CDCl_3$); 6.67-6.54 (m, 3H), 6.36 (d, 2H, *J* = 17.3 Hz), 5.77 (br s, 1H), 5.58 (d, 2H, *J* = 10.6 Hz), 4.27-4.22 (m, 2H), 3.79-3.63 (m, 8H), 2.48-2.45 (m, 1H) ppm; **δ_C** (101 MHz, $CDCl_3$); 163.8, 162.6, 136.0, 121.5, 105.5, 79.6, 74.7, 70.3, 70.1, 69.2, 58.4, 41.2 ppm; **HRMS** (ESI) calculated for $[C_{15}H_{20}N_3O_2]^+$ 274.1550 *m/z* found 274.1544 $[M+H]^+$

2-(2-(2-Azidoethoxy)ethoxy)acetic acid (6)

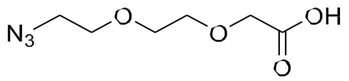

2-(2-(2-azidoethoxy)ethoxy)acetate cyclohexanaminium salt (900 mg, 3.14 mmol) was dissolved in 1 mL of H_2O and acidified to pH 2 with conc HCl. The aqueous phase was diluted with water and extracted with 10% isopropanol in CH_2Cl_2 (4 x 10 mL). The combined organic extracts were combined and the solvent removed in vacuo to yield **6** (590 mg, 3.14 mmol, quantitative) as a brown oil.

R_f 0.1 (10% EtOAc/PE 40-60); **ν_{max}** 2918 (m), 2101 (str), 1735 (str), 1441 (m), 1346 (m), 1285 (m), 1145 (str), 1150 (str), 932 (w), 854 (w), 668 (w) cm^{-1}; **δ_H** (400 MHz, $CDCl_3$) 4.21 (s, 2H), 3.79-3.77 (m, 2H), 3.74-3.69 (m, 4H), 3.44-3.42 (m, 2H) ppm; **δ_C** (101 MHz, $CDCl_3$) 173.1, 71.4, 71.3, 70.5, 70.5, 70.2, 68.7 ppm.

NMR spectra

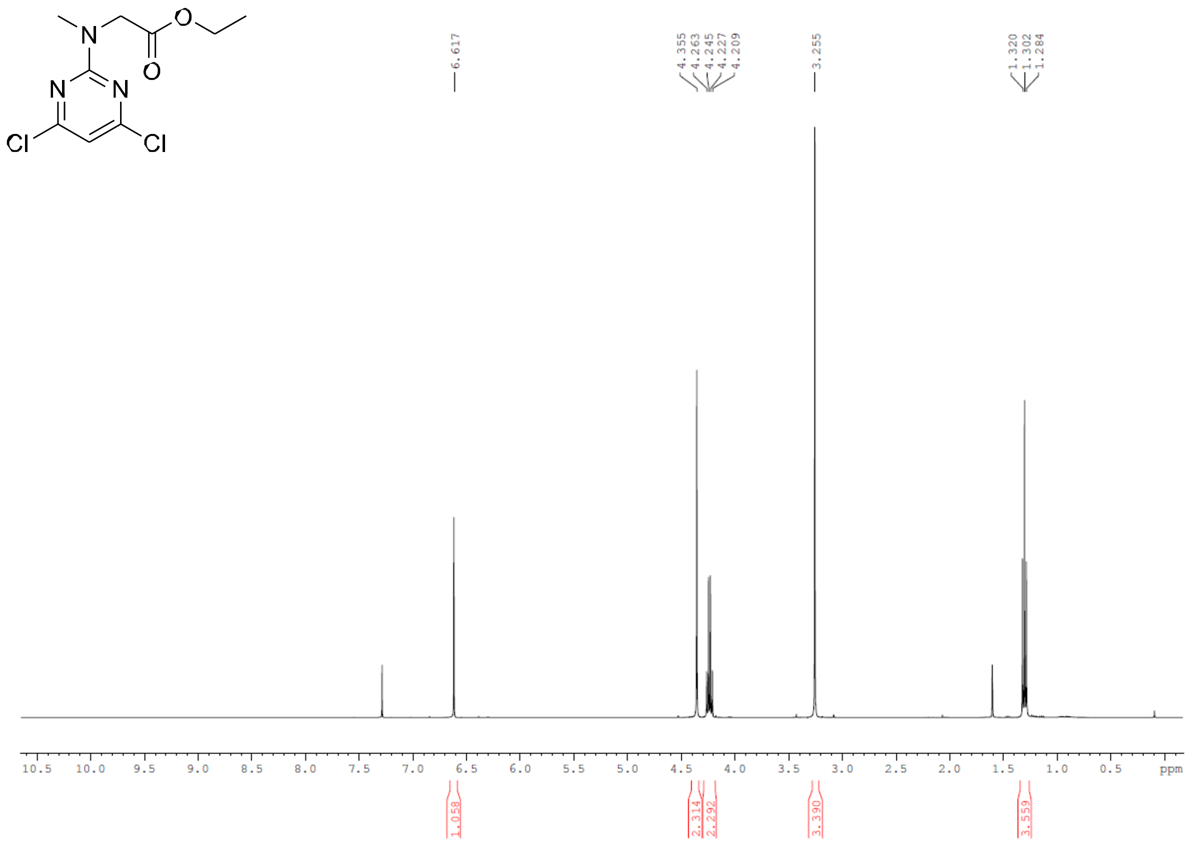
N
O
O
N
N
Cl
Cl
6.617
4.355
4.263
4.245
4.227
4.209
3.255
1.320
1.302
1.284
ppm
1.058
2.314
2.292
3.390
3.559

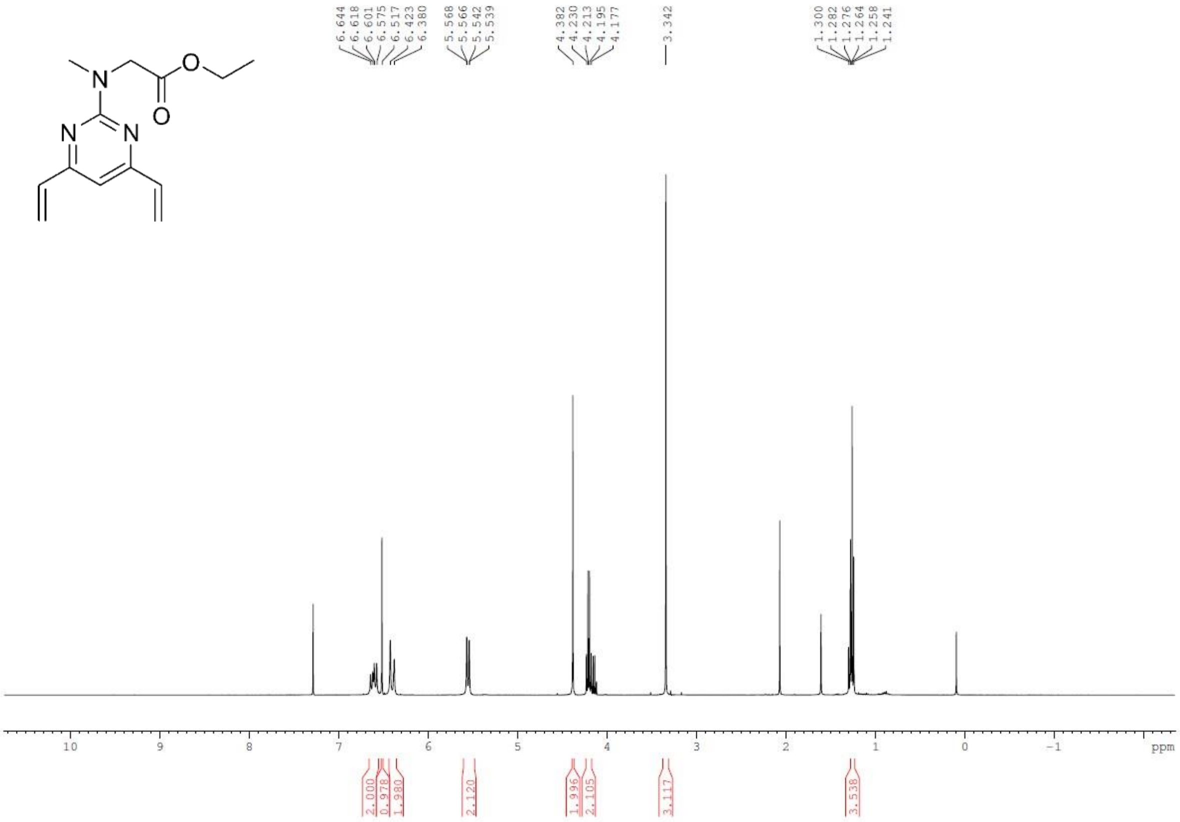
N
O
O
N
N
6.644
6.618
6.601
6.575
6.517
6.423
6.380
5.568
5.566
5.542
5.539
4.382
4.230
4.213
4.195
4.177
3.342
1.300
1.282
1.276
1.264
1.258
1.241
ppm
2.000
0.978
1.980
2.120
1.996
2.105
3.117
3.538

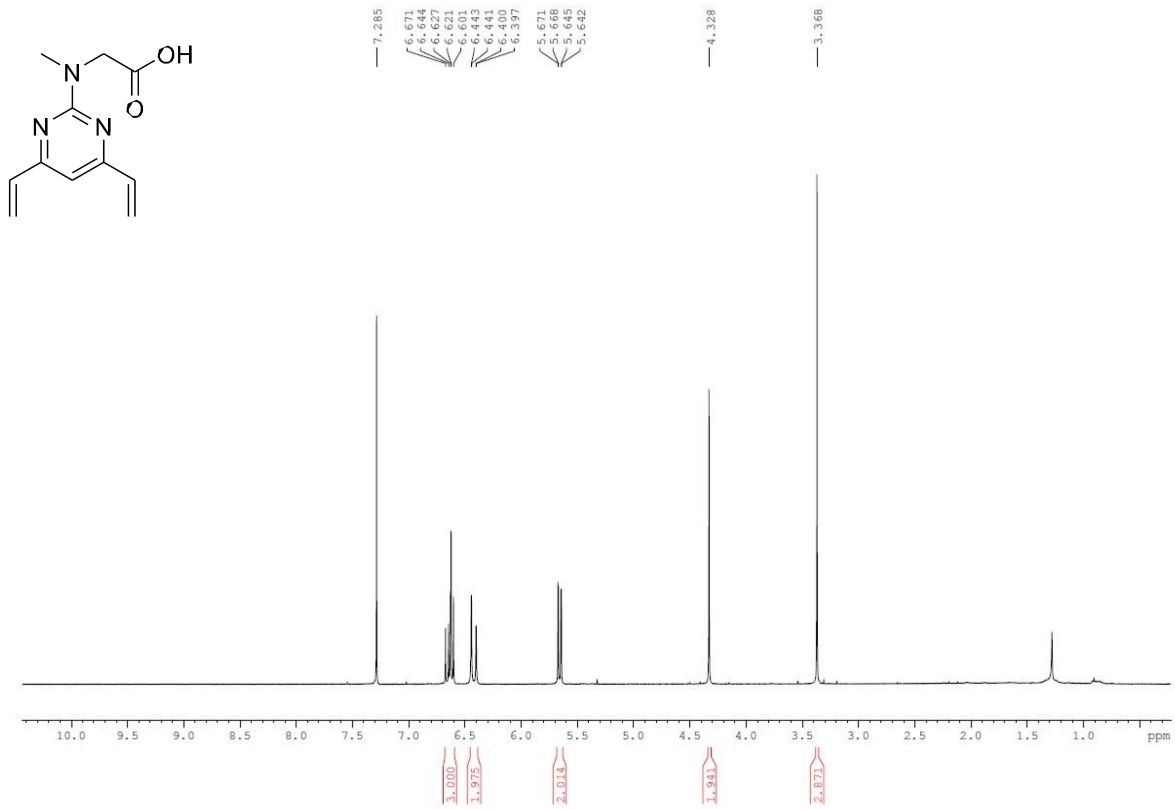
OH
N
O
N
N
7.285
6.671
6.644
6.627
6.621
6.601
6.443
6.441
6.400
6.397
5.671
5.668
5.645
5.642
4.328
3.368
10.0 9.5 9.0 8.5 8.0 7.5 7.0 6.5 6.0 5.5 5.0 4.5 4.0 3.5 3.0 2.5 2.0 1.5 1.0 ppm
3.000
1.975
2.014
1.941
2.871

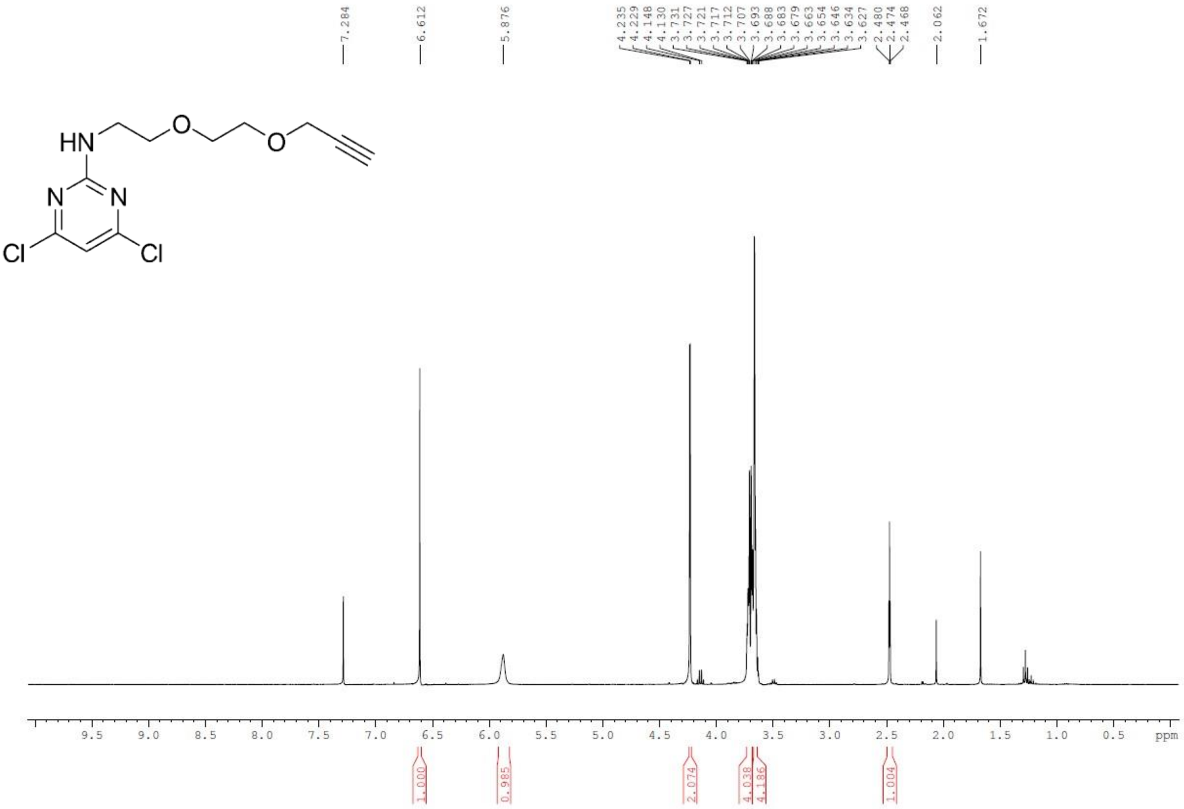
7.284
6.612
5.876
4.235 4.229 4.148 4.130 3.731 3.727 3.721 3.717 3.712 3.707 3.693 3.688 3.683 3.679 3.663 3.654 3.646 3.634 3.627
2.480 2.474 2.468
2.062
1.672
HN
O
O
N
N
Cl
Cl
9.5 9.0 8.5 8.0 7.5 7.0 6.5 6.0 5.5 5.0 4.5 4.0 3.5 3.0 2.5 2.0 1.5 1.0 0.5 ppm
1.000
0.985
2.074
4.038
4.186
1.004

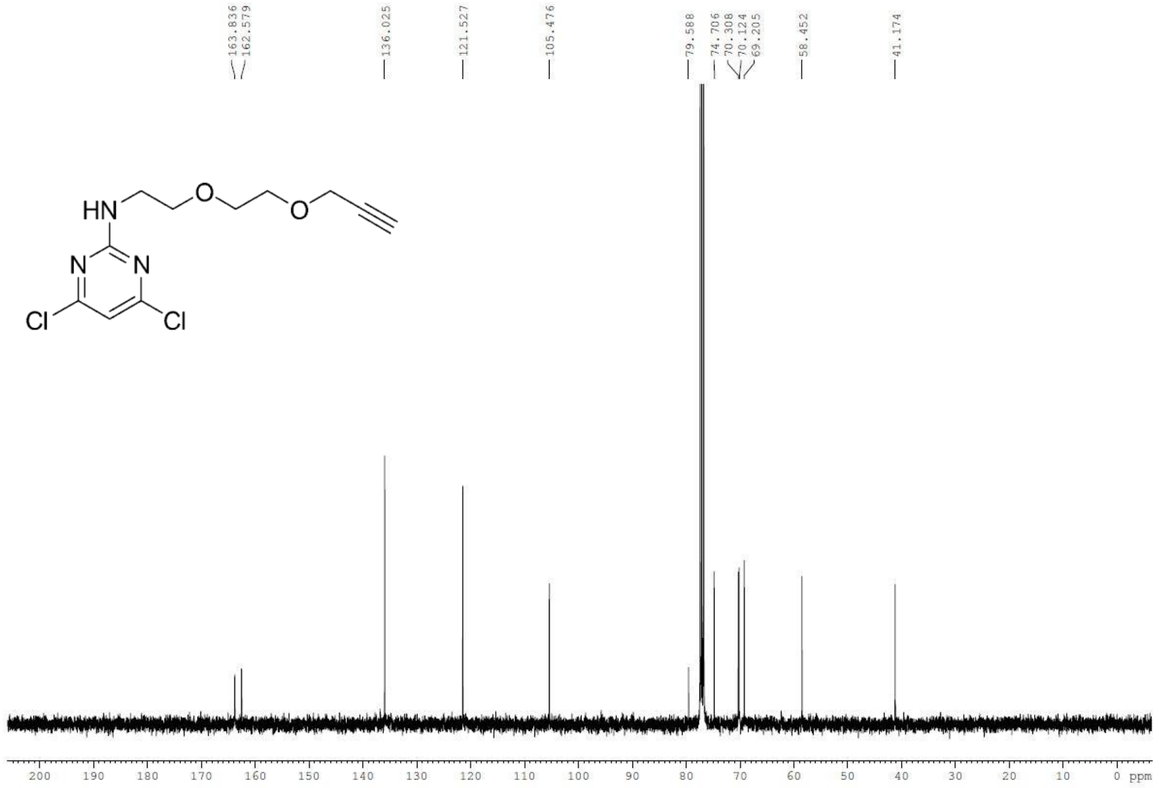
163.836
162.579
136.025
121.527
105.476
79.588
74.706
70.308
70.124
69.205
58.452
41.174
HN
O
O
N
N
Cl
Cl
200 190 180 170 160 150 140 130 120 110 100 90 80 70 60 50 40 30 20 10 0 ppm

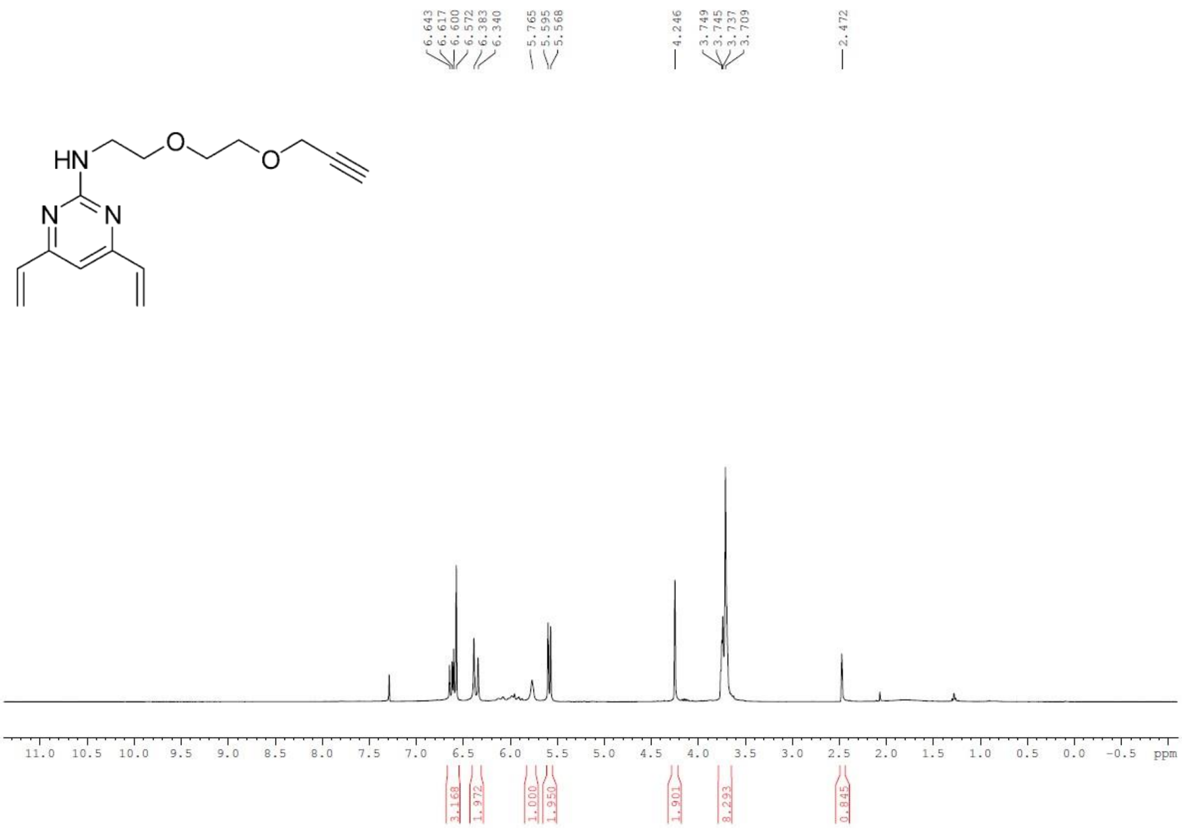

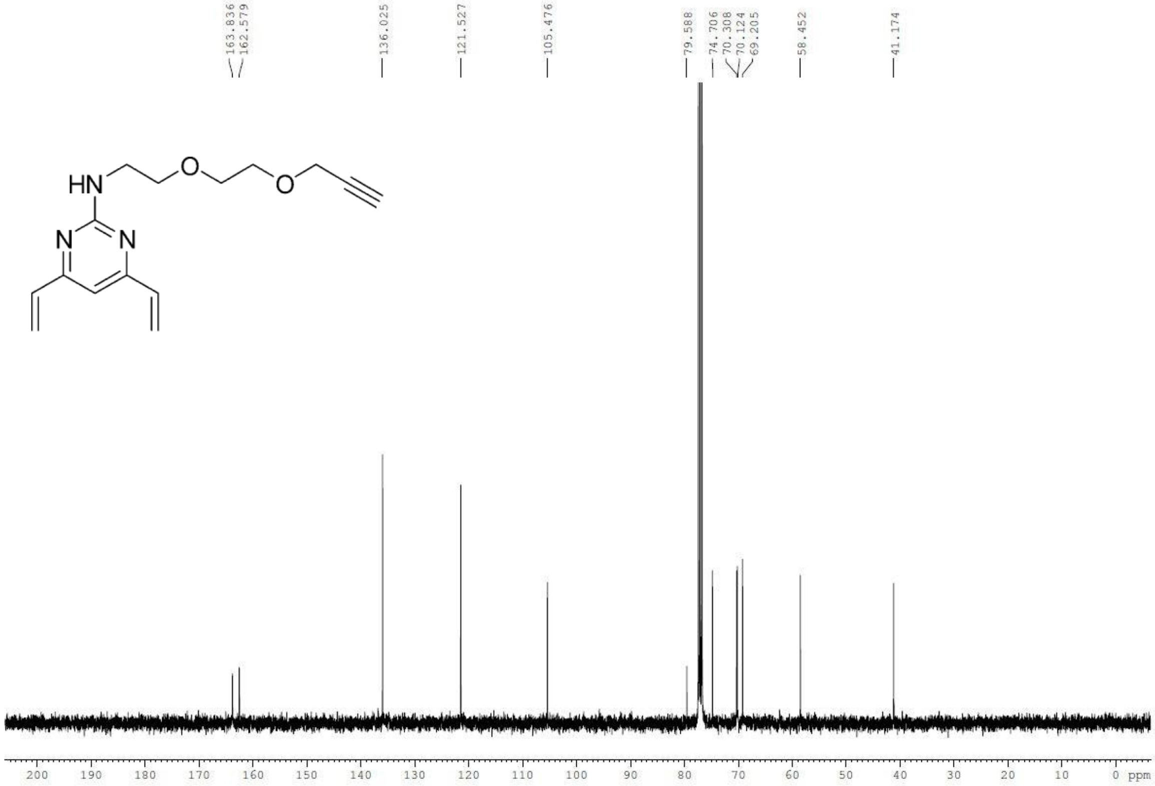

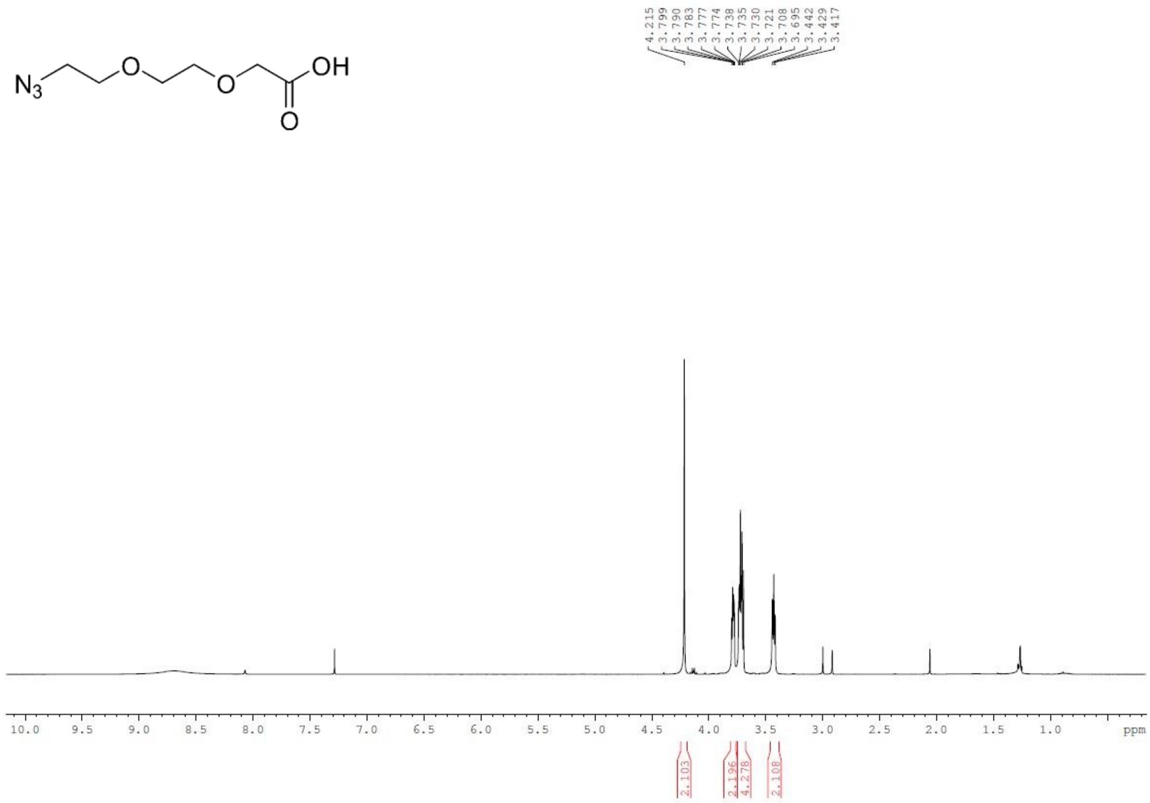
N3 O O OH O
4.215
3.799
3.790
3.783
3.777
3.774
3.738
3.735
3.730
3.721
3.708
3.695
3.442
3.429
3.417
10.0 9.5 9.0 8.5 8.0 7.5 7.0 6.5 6.0 5.5 5.0 4.5 4.0 3.5 3.0 2.5 2.0 1.5 1.0 ppm
2.103
2.196
4.278
2.108

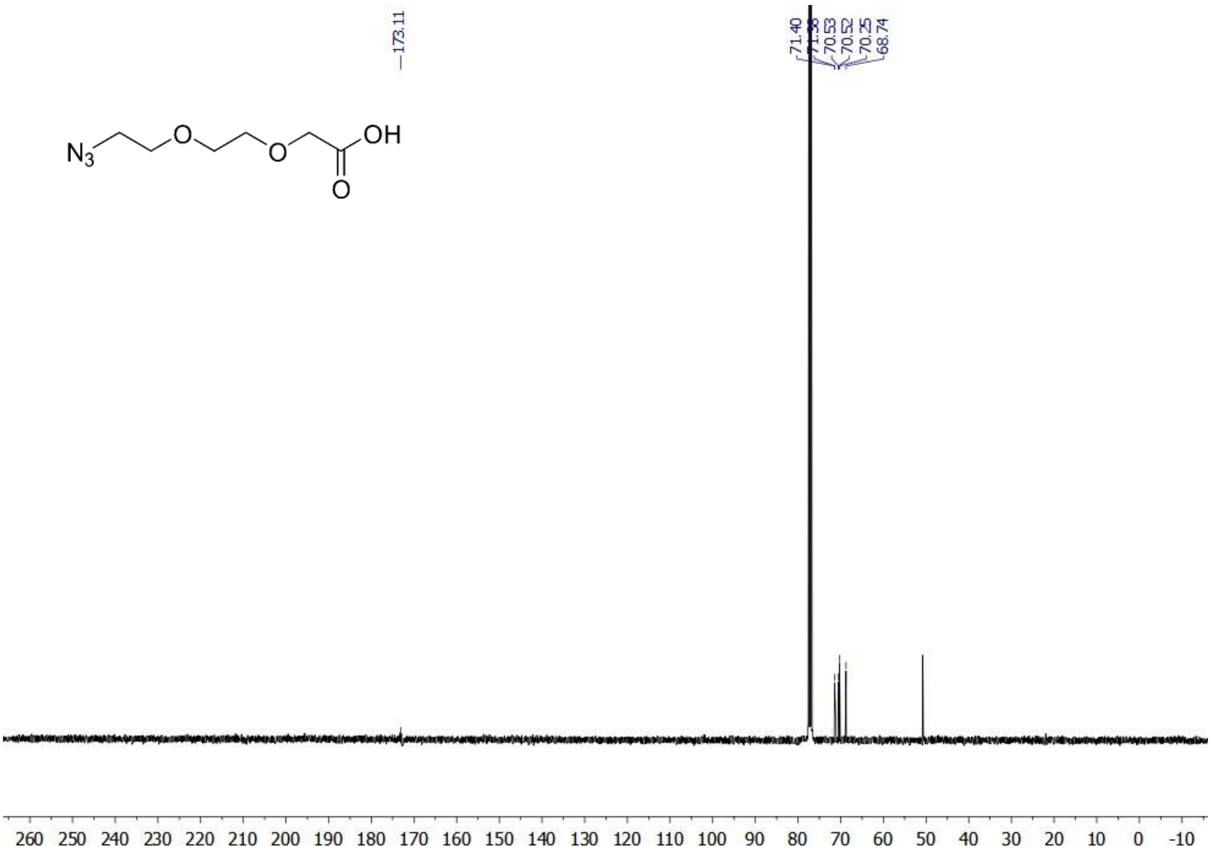
173.11
71.40
70.53
70.52
70.25
68.74
N3 O O OH O
260 250 240 230 220 210 200 190 180 170 160 150 140 130 120 110 100 90 80 70 60 50 40 30 20 10 0 -10

Peptide synthesis

Manual peptide synthesis

Manual peptide synthesis was performed on Merck LL MHBA Rink amide resin (0.51 mmol/g, 1 equiv). Couplings were carried out by adding HATU (4 equiv) to a solution of the Fmoc-protected amino acid (4 equiv) in DMF (~0.4 M). After 10 seconds, DIPEA (8 equiv) was added to the mixture. This pre-activated mixture was then added to the resin in DMF and shaken for 3 minutes. The coupling time was extended in the case of amide coupling when substrates other than natural amino acids were used (30 minutes). The side chain protecting groups used were: ^{t}Bu for Asp, Glu, Thr; Boc for Lys; Pbf for Arg; Trt for Asn, Gln. Fmoc-Lys(iddve)-OH was used for conjugation of stearic acid with Lys.

Fmoc deprotection was carried out with 20% piperidine in 0.1 M HOBt in DMF (3 x 3 minutes).

N-terminal capping with FITC and Ac_2O (2 equiv) was achieved using DIPEA (4 equiv) in CH_2Cl_2 overnight (FITC) or for 1 hour (Ac_2O).

On-resin attachment of stearic acid *via* Lys was achieved by orthogonal deprotection of the Lys(ddve) with 5% NH_2NH_2 in DMF (2 x 10 minutes) followed by standard amide coupling as described before.

Completion of amide couplings and Fmoc deprotection was determined by a chloranil test, in which acetaldehyde (200 µL) and a saturated solution of chloranil in toluene (50 µL) were added to a small amount of resin swelled in CH_2Cl_2. After 10 seconds shaking at rt, no change in colour indicated complete coupling, whilst green colouration of the resin indicated presence of a free amine. Incomplete couplings were submitted to a second round of coupling.

Side chain deprotection and cleavage from the resin was achieved with TFA containing 2.5% TIPS and 2.5% H_2O for 3 hours at rt. In case of methionine and cysteine-containing peptides, cleavage was achieved with TFA containing 5% EDT, 5% H_2O and 2.5% TIPS. After cleavage, the mixture was filtered through a sintered funnel, the beads washed with MeOH and the filtrate was concentrated under a stream of N_2. The crude residue was triturated with cold Et_2O before purification by preparative HPLC. **H** was not triturated with Et_2O due to its lipophilic nature.

Automated Fmoc solid-phase peptide synthesis

Automated SPPS was carried out on solid-phase using a Fmoc-protecting group strategy on a CEM Liberty Blue® automated microwave peptide synthesiser.

Automated peptide synthesis was performed using Merck LL MHBA Rink Amide resin (0.51 mmol/g, 1 equiv). All peptide couplings were performed with Fmoc-protected amino acids (5 equiv), Oxyma pure

(10 equiv) and DIC (5 equiv) in DMF. Arg was coupled using double couplings for 15 min each without microwave irradiation. All other amino acids were coupled with 25 W power at 75°C over 15 min.

Fmoc deprotection was achieved with a solution of 20% piperidine in DMF, using 45 W power at 75°C over 3 min. N-terminal capping, cleavage and HPLC purification of peptides were carried out as previously described for manual SPPS (general method 10).

DVP stapling reaction

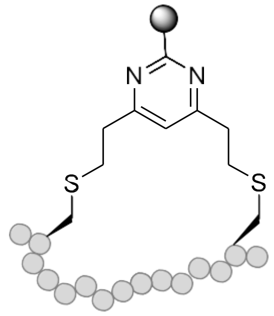

The linear peptide (1 equiv.) was dissolved in 50 mM NaPi buffer pH 8.0 (containing 5% of DMF) to a final concentration of 2 mg/mL. The staple (1.1 equiv) was added and the reaction shaken at rt for 1 h. Upon completion as monitored by analytical HPLC, the crude reaction was lyophilised and purified on a preparatory HPLC to yield the cyclised peptide.
Adapted from S. Walsh *et al*.[1]

DCA stapling reaction

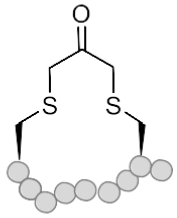

To a solution of the linear peptide (1 equiv) in 50 mM NaPi buffer pH 8.0 was added TCEP·HCl (1.5 equiv) and the solution stirred for 1 h. Subsequently, a solution of dichloroacetone (3 equiv) in DMF (600 uL/mg of dichloroacetone) was added and the solution shaken for 4 h. The solvent was removed under a stream of N_2 and the crude reaction mixture dissolved in DMSO before being purified on a preparatory HPLC to yield the pure peptide.

Adapted from Dawson *et al*.[3]

[3] Acetone-Linked Peptides: A Convergent Approach for Peptide Macrocyclization and Labeling, N. Assem, D. J. Ferreira, D.W. Wolan, P. E Dawson, *Angew. Chem Int Ed*, **2015**, 20, 54, 8665-8668

Dibromobenzene stapling reaction

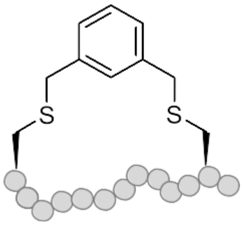

To a solution of the peptide (1 equiv) in 50 mM NaPi buffer pH 8.0 (2mg/mL) was added TCEP·HCl (1.5 equiv) and the solution stirred at rt for 1 h. Subsequently, a solution of 1,3-bis(bromomethyl)benzene (3 equiv) in DMF (6 mM) was added and the solution shaken for 4 h. The solvent was removed under a stream of N_2 and the crude reaction mixture dissolved in DMSO before being purified on a preparatory HPLC to yield the pure peptide.

Adapted from Timmerman *et al*.[4]

Diiodometane stapling reaction

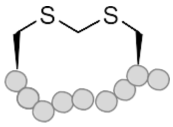

To a solution of the linear peptide (1 equiv) in H_2O (2 mg/mL) was added a solution of TCEP·HCl (1.5 equiv) and K_2CO_3 (3 equiv) in H_2O (100 uL per mg of TCEP). The reaction was monitored by LCMS. Upon completion of this step, a solution of NEt_3 (10 equiv) in THF (20 uL/uL of NEt_3) and diiodomethane (8 equiv) in THF (20 uL/mg of diiodomethane) were added sequentially. The reaction mixture was stirred at rt for 4 h. Solvent was removed under N_2 and the crude reaction mixture dissolved in DMSO prior to purification on a preparatory HPLC to obtain the pure peptide.

Adapted from Kourra *et al*.[5]

[4] Synthesis of water-soluble scaffolds for peptide cyclisation, labelling and ligation. L. E. J. Smeenk, N. Dailly, H. Hiemstra, J. H. Van Maarseveen, P. Timmerman, *Org. Lett.* **2012**, *14*, 1194.

[5] Converting disulfide bridges in native peptides to stable methylene thioacetals. C. M. B.K Kourra, N. Cramer, *Chem Sci*. **2016**, 7, 7007-7012.

Copper-catalysed azido-alkyne click (CuAAC)

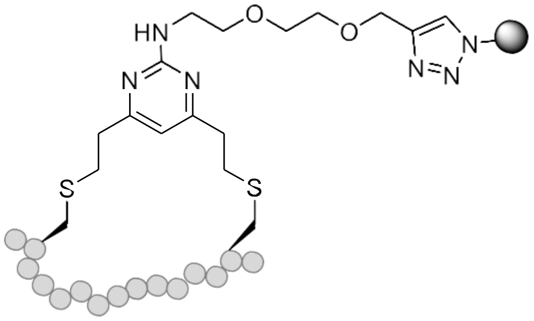

A solution of azido-containing linker (1 equiv) and alkyne-containing stapled peptide (1 equiv) in 1:1 ^{t}BuOH/H_2O (0.8 mL/mg peptide) was degassed with N_2 for 15 min, followed by the addition of $CuSO_4$ ·5 H_2O (1 equiv), THPTA (1 equiv), and sodium ascorbate (3 equiv). Peptides bearing sulfur atoms required $CuSO_4$ · 5 H_2O (2 equiv), THPTA (2 equiv), and sodium ascorbate (6 equiv). All the reactions were stirred under N_2 and monitored by LCMS. When no starting material could be detected by LCMS, the reaction mixture was diluted with H_2O and lyophilised prior to purification.

Adapted from J. Iegre *et al*.[6]

Synthesis of **G** and **H** stapled peptides

Synthesis of **G** and **H** stapled peptides occurred following the generic scheme presented in Scheme S2.

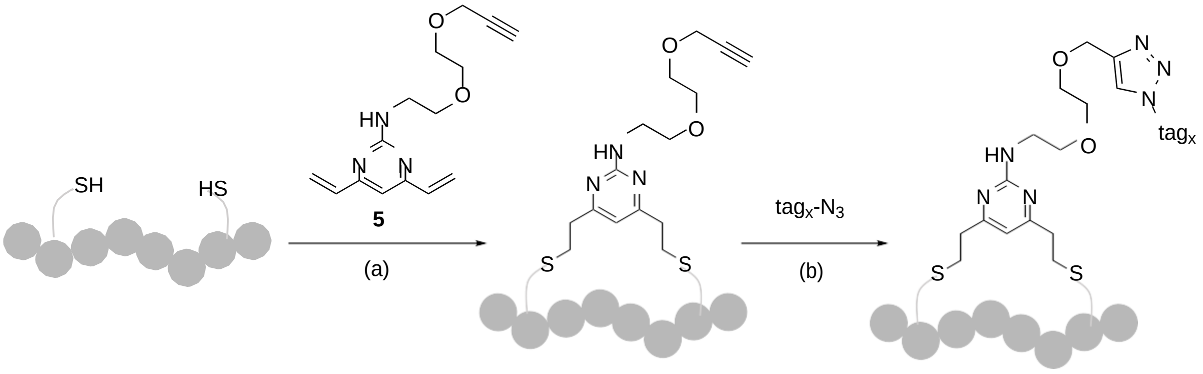

*Scheme S2 - Generic synthetic route to **G** and **H** functionalised stapled peptides. (a) performed following the DVP stapling reaction conditions; (b) Peformed following the CuAAC conditions. In G functionalised stapled peptides, tag$_x$ = G synthesised as described below; in H functionalisd stapled peptides, tag$_x$ = H synthesised as described below.*

[6] Efficient development of stable and highly functionalised peptides targeting the CK2a/CK2b protein-protein interaction, J. Iegre, P. Brear, D. J. Baker, Y. S. Tan, E. L. Atkinson, H. Sore, D. H. O'Donovan, C. Verma, M. Hyvonen, D. R. Spring, *Chem. Sci.* **2019**, *10*, 5056-5063

Staple **H** synthesis

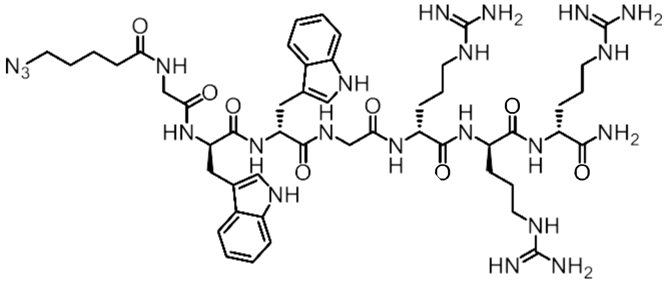

tag1 (APA-GWWGrrr)

NHFmoc-GWWGrrr-CONH$_2$ (300 mg of starting resin) was synthetized on Rink amide resin (0.308 mmol/g loading) according to the abovementioned standard SPPS procedure with use of Fmoc-Trp(Boc)-OH, Fmoc-Gly-OH and Fmoc-(D)-Arg(Pbf)-OH amino acids. Fmoc protecting group was cleaved by 20% piperidine in DMF (2 x 10 min), followed by wash with DMF (2x) and CH_2Cl_2 (5x). Then, solution of 5-azidopentanoic acid (APA) (50 µL, 0.4 mmol), HATU (152 mg, 0.4 mmol) and DIPEA (132 µL, 0.8 mmol) in DMF (2 mL) was added to the resin and the reaction slurry was shaken at ambient temperature for 16 h, followed by wash with DMF (2x) and CH_2Cl_2 (5x). The prepared **H** was cleaved from the resin by abovementioned procedure for 3 h, followed by HPLC purification.

Staple **G** synthesis

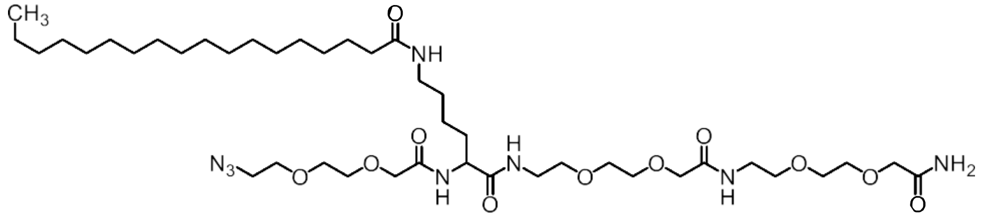

tag2

G (250 mg of starting resin) was prepared by abovementioned procedure for peptide coupling on Rink amide resin (0.308 mmol/g loading) using (i, ii) 8-(Fmoc-amino)-3,6-dioxaoctanoic acid (0.3M solution in DMF), (iii) Fmoc-Lys(ivDde)-OH (0.3M solution in DMF), (iv) 2-(2-(2-azidoethoxy)ethoxy)acetic acid (0.2M solution in DMF) and (v) stearic acid (0.2M solution in DMF), HATU and DIPEA. Reaction time or each step was 6 to 16 h. ivDde protecting group was cleaved in 10% hydrazine hydrate in DMF for 3 h. The prepared tag2 was cleaved from the resin by abovementioned procedure for 3 h, followed by HPLC purification.

Peptide characterization

*Table S1 - Characterisation of the peptides presented in this work. *Measured on a 5-95% gradient*

Peptide sequence	Code	Purity	Expected mass	Mass found and species	HPLC Rt (10-60% ACN in H_2O over 15 min)
LQLDPETGEFL	**Native**	96%	1302.4	1303.1 [M+H$^+$]	10.23
CQLDPETGECL	**P1**	85%	1248.4	1248.7 [M+H$^+$]	7.09
C$_A$QLDPETGEC$_A$L	**P1-A**	88%	1260.4	1260.6 [M+H$^+$]	7.11
C$_B$QLDPETGEC$_B$L	**P1-B**	>99%	1302.4	1303.1 [M+H$^+$]	6.83
C$_C$QLDPETGEC$_C$L	**P1-C**	99%	1350.5	1352.9 [M+H$^+$]	8.09
C$_D$QLDPETGEC$_D$L	**P1-D**	99%	1467.6	1468.9 [M+H$^+$]	7.23
LCEGTEPDCQL	**P1-inversed**	90%	1247.5	1249.2 [M+H$^+$]	8.09
LQCDPETGECL	**P2**	94%	1248.4	1249.7 [M+H$^+$]	7.20
LQC$_A$DPETGEC$_A$L	**P2-A**	98%	1260.4	1258.9 [M-H$^+$]	7.21
LQC$_B$DPETGEC$_B$L	**P2-B**	96%	1302.4	1302.2 [M$^+$]	8.26
LQC$_C$DPETGEC$_C$L	**P2-C**	85%	1350.5	1351.1 [M+H$^+$]	8.19
LQC$_D$DPETGEC$_D$L	**P2-D**	98%	1467.6	1468.0 [M+H$^+$]	7.36
C$_D$LQLDPETGEC$_D$L	**P3-D**	97%	1580.81	1579.2 [M-H$^+$]	9.52
AzaLQLDPETGEAzaL	**P3Aza-E**	98%	1549.6	1550.2 [M+H$^+$]	8.83
CLQLDPETGECL	**P3**	78%	1361.5	1360 [M-H$^+$]	9.48
C$_F$LQLDPETGEC$_F$L	**P3-F**	93%	1608.8	1609.9 [M+H$^+$]	10.26
C$_F$LQLD$_{(OMe)}$PE$_{(OMe)}$TGE$_{(OMe)}$C$_F$L	**P7-F**	99%	1650.9	1651.2 [M$^+$]	15.73
C$_F$LQLDPE$_{(OMe)}$TGE$_{(OMe)}$C$_F$L	**P6-F**	96%	1636.9	1636.0 [M-H$^+$]	9.02
C$_F$LQLD$_{(OMe)}$PETE$_{(OMe)}$C$_F$L	**P4-F**	98%	1636.9	1636.7 [M$^+$]	11.22
C$_F$LQLD$_{(OMe)}$PE$_{(OMe)}$TGEC$_F$L	**P5-F**	88%	1636.9	1639.0 [M-H$^+$]	11.37
CLQLD$_{(OMe)}$PE$_{(OMe)}$TGE$_{(OMe)}$CL	**P7**	87%	1403.6	1404.5 [M+H$^+$]	8.91
CLQLDPE$_{(OMe)}$TGE$_{(OMe)}$CL	**P6**	95%	1389.6	1390.1 [M+H$^+$]	9.40
CLQLD$_{(OMe)}$PETGE$_{(OMe)}$CL	**P4**	96%	1389.6	1389.9 [M$^+$]	9.27
CLQLD$_{(OMe)}$PE$_{(OMe)}$TGECL	**P5**	95%	1389.6	1389.8 [M$^+$]	9.40
LCEGTEPDLQC	**P2 inversed**	>99%	1248.4	1249.2 [M+H$^+$]	8.01
**6**-PEG$_2$-K(C$_{18}$)-PEG$_2$-PEG$_2$-NH$_2$	**H**	99%	1017	1017.0 [M$^+$]	15.85*
C$_5$LQLDPETGEC$_5$L clicked with **H**	**P3-H**	96%	2652.0	1327.4 [M+2H$^+$]	12.59*
C$_5$LQLDPETGEC$_5$L clicked with **G**	**P3-G**	90%	2286.9	2289.2 [M+H$^+$]	10.38
N$_3$-Apa-GWWG-(D)R(D)R(D)R-NH$_2$	**G**	88%	1110.6	1111.9 [M+H$^+$]	9.07
CDPETGECL	**P8**	98%	1007.1	1005.4[M-2H$^+$]	5.79
C$_5$DPETGEC$_5$L	**P8-5**	54%	1280.4	1278.5[M-2H$^+$]	7.53
C$_5$DPETGEC$_5$L clicked with **G**	**P8-G**	93%	2377.7	1189.7[M+H$^+$]	7.61
C$_5$DPETGEC$_5$L clicked with **H**	**P8-H**	97%	2153.5	2152.4 [M-H$^+$]	15.39*

Table S2 - HPLC trace of peptides

Native	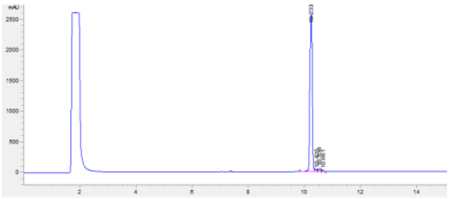	P1	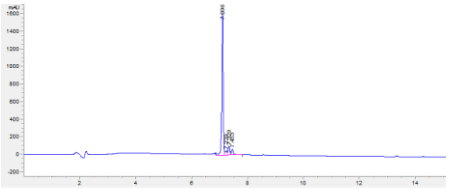
P1-A	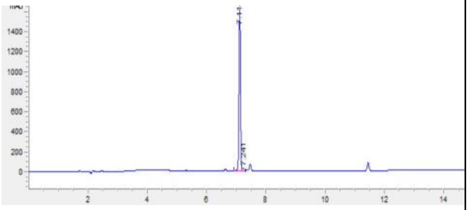	P1-B	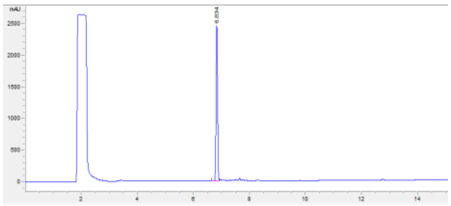
P1-C	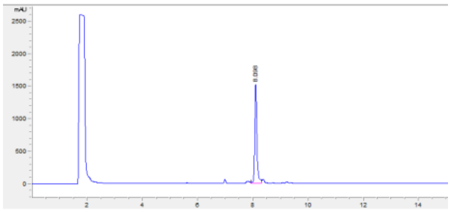	P1-D	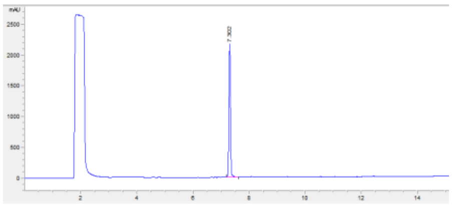
P1 inverse	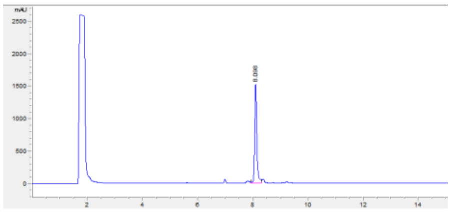	P2	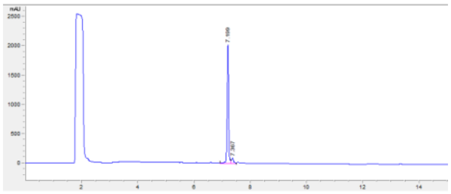
P2-A	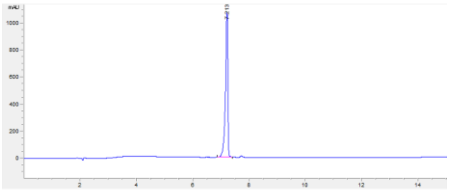	P2-B	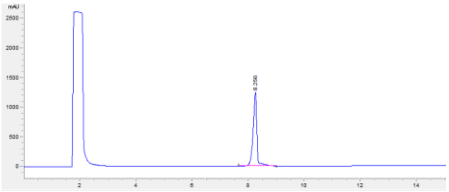
P2-C	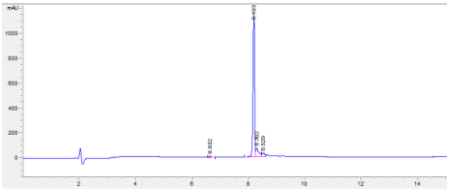	P2-D	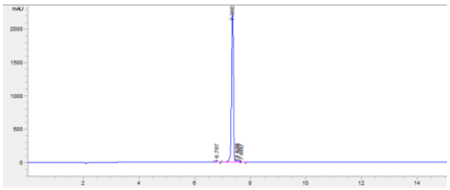
P3-D	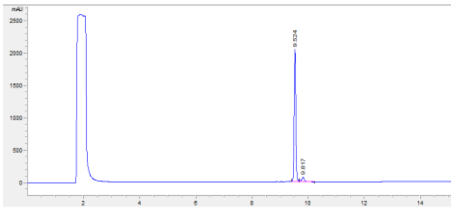	P3-E	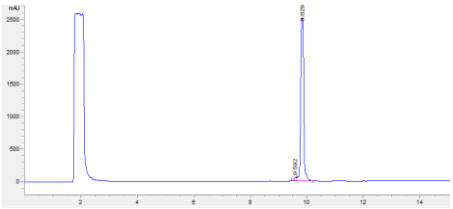

P3

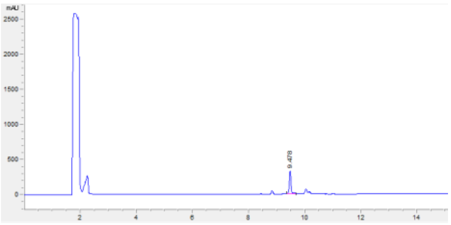

P7-F

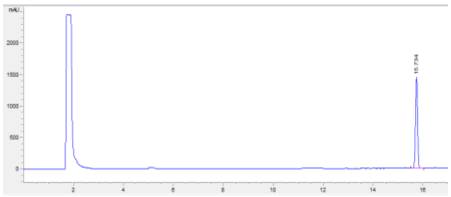

P6-F

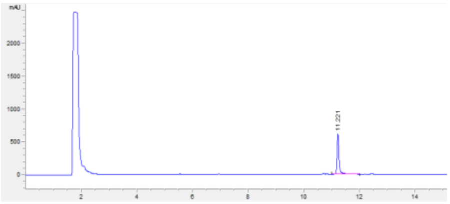

P8

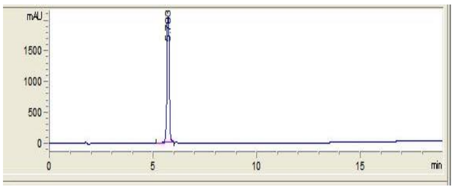

H

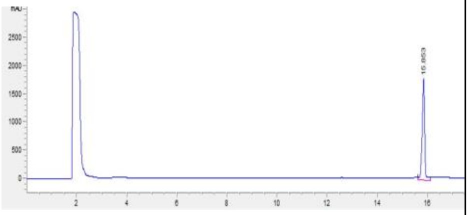

P3-G

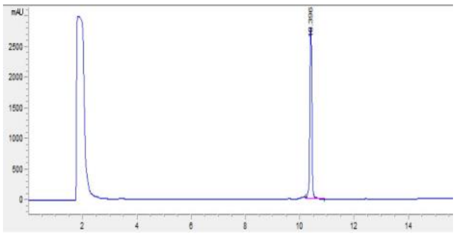

P8-H

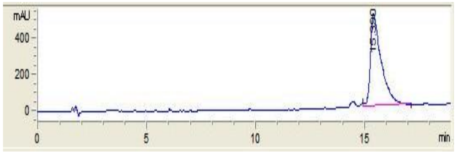

P3-F

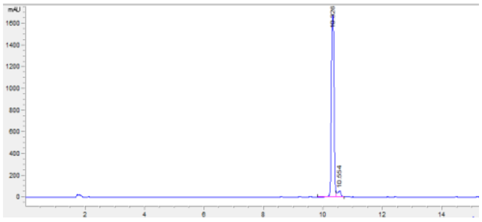

P5-F

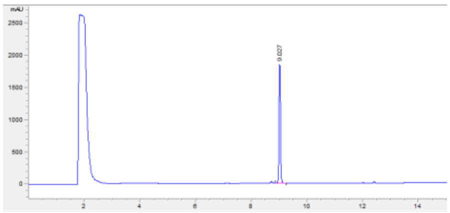

P4-F

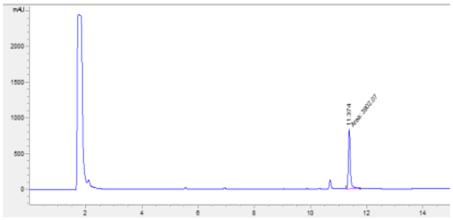

G

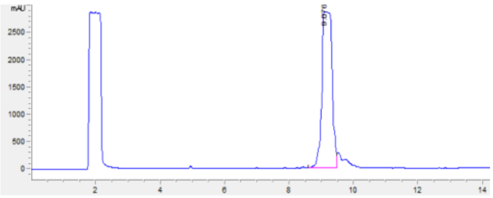

P3-H

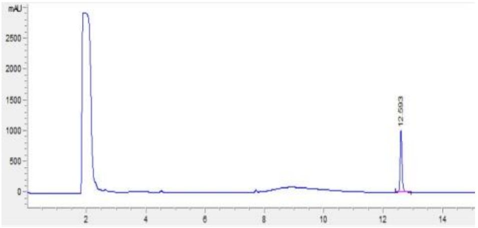

P8-G

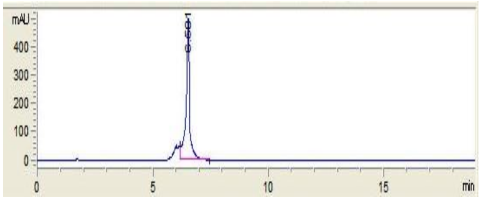

Biophysics Experimental

Surface Plasmon Resonance (SPR)

Peptide affinities to the Kelch domain of Keap1 is determined in a direct binding assay using 8K surface plasmon resonance (SPR) biosensor (Cytiva) at 20°C. Immobilization of Keap1 Kelch domain on a CM5 sensor chip (Cytiva) is performed using standard amine coupling procedure. The surface is washed with 10mM NaOH, 1M NaCl before activated with EDC/NHS (Cytiva), followed by immobilization of human Keap1 Kelch domain (A321-T609) fused with N-terminal 6x His-tag[7] (in 10 mM MES, pH=6.4). Finally, the surface is deactivated by Ethanolamine. Immobilization levels are typically 5000-6000 RU. The reference spot is treated as described, omitting the injection of Keap1. Compound concentration series are injected over the immobilized protein in increasing concentrations using single cycle kinetics (SCK) in running buffer (10 mM HEPES, 150 mM NaCl, 0.05% Tween20, 1 mM TCEP, pH 7.4). A 1:1 Langmuir interaction model is fitted to the experimental traces, enabling determination of k_{on}, k_{off} and K_d.

Keap1-Nrf2 TR-FRET Assay

In the time-resolved Förster resonance energy transfer (TR-FRET) assay[8] the IC_{50} of inhibitors which are able to inhibit the interaction between the Keap1-Kelch domain and a Nrf2 derived ETGE peptide are determined. The TR-FRET is measured between a Tb-labelled mAb (Anti—6-His, Tb cryptate Gold, CisBio 61H12TLB) that binds to Keap 1 Kelch domain and d2-labelled SA-biotin-peptide.

The assay was was performed in 384-well white low-volume, medium binding plates (Greiner Bio One, Austria) in 20 mM HEPES pH 7.5, 150 mM NaCl, 2 mM GSH (Sigma-Aldrich, USA),1mM EDTA (Sigma-Aldrich, USA), and 0.01% Pluronic F127 (MilliporeSigma, USA), with a final reaction volume of 10 µL.

To determine the ability of synthesized peptide to inhibit PPI between the Keap1 Kelch domain and Nrf2, 1nM purified human Keap1 Kelch domain (A321-T609) fused with N-terminal 6x His-tag, 5 nM biotinylated Nrf2 peptide (KKKKAFFAQLQLDEETGEFL, Genescript, USA), d2-labeled streptavidin (Revvity, USA), and 0.1 mM anti-His monoclonal antibody tagged with terbium cryptate (Revvity, USA), were incubated in the presence of varying concentrations of investigated peptides for 3 h at room temperature.

[7] Li X, Zhang D, Hannink M, Beamer LJ. Crystallization and initial crystallographic analysis of the Kelch domain from human Keap1. Acta Crystallogr D Biol Crystallogr. 2004 Dec;60(Pt 12 Pt 2):2346-8. doi: 10.1107/S0907444904024825. Epub 2004 Nov 26. PMID: 15583386.
[8] S. Lee, D. Ali Abed, L. J. Beamer, L. Hu, Development of a Homogeneous Time-Resolved Fluorescence Resonance Energy Transfer (TR-FRET) Assay for the Inhibition of Keap1–Nrf2 Protein–Protein Interaction, SLAS Discovery, 26, 1, 2021, Pages 100-112, ISSN 2472-5552,https://doi.org/10.1177/2472555220935816.

Following the incubation, the PHERAstar FSX (Revvity, USA) microplate reader was used to measure the acceptor and donor emissions at 665 nm and 620 nm, respectively. The acceptor/donor ratio was used to calculate relative energy transfer. Data was normalized by two-point normalization using neutral and inhibitor controls (neutral control: DMSO, inhibitor control: 0.1nM 2-[[4-[carboxymethyl-(4-methoxyphenyl)sulfonylamino]naphthalen-1-yl]-(4-methoxyphenyl)sulfonylamino]acetic acid; Jiang Z-Y. *et al. J. Med. Chem.* **2014**, *57*, 2735-2745). AZ13761168). Data was fitted using four parameter logistic fit and IC_{50} values were calculated using Genedata screener software (Genedata, Switzerland).

X-ray crystallography

Crystals were grown at 20c in sitting drops using 0.2µl of mouse Keap1 protein[9] (15mg/ml) and 0.2µl of well solution (0.7-0.9M Lithium Sulfate, 0.5-0.7M Ammonium sulfate and 0.1M Sodium Citrate pH5.9). Prior to soaking trials 1µl of compound **P3-F** and **P8-F** (10mM stock) was dried in on a cover slip and thereafter 2µl of soaking solution (33% PEG4000, 0.1M BisTris pH7) was added to form a 5mM soak drop. Crystals were soaked overnight and then frozen in liquid nitrogen using soaking solution supplemented with 20% Glycerol as cryo protectant prior to data collection.

Data was collected at the BioMAX beamline at MAX IV, in Lund, Sweden. Data was processed using autoPROC[10] with the STARANISO[11] option. Initial processing suggested that the crystals belong to space group $P6_2$. Molecular replacement using the program PHASER[12] with the search model 5FZN[13] identified one molecule in the asymmetric unit. The model was refined using the program autoBUSTER[14] and subsequently rebuilt manually in Coot[15]. The peptidic ligand was readily identified and could be built into difference density. Ligand restraints were generated with the program Grade[16].

---

9 *J. Med. Chem.* 2019, 62, 9, 4683–4702, 2019 https://doi.org/10.1021/acs.jmedchem.9b00279

10 Data processing and analysis with the autoPROC toolbox , Vonrhein, C.; Flensburg, C.; Keller, P.; Sharff, A.; Smart, O.; Paciorek, W.; Womack, T.; Bricogne, G. *Acta Crystallogr. D. Biol. Crystallogr.* **2011**, *67* (Pt 4), 293-302.

11 Tickle, I.J., Flensburg, C., Keller, P., Paciorek, W., Sharff, A., Vonrhein, C., Bricogne, G. **2018** STARANISO. Cambridge, United Kingdom: Global Phasing Ltd.

12 McCoy, A.J., Grosse-Kunstleve, R.W., Adams, P.D., Winn, M.D., Storoni, L.C., Read, R.J. Phaser crystallographic software. J. Appl. Cryst. 2007, 40:658-674.

13 Monoacidic Inhibitors of the Kelch-like ECH-Associated Protein 1: Nuclear Factor Erythroid 2-Related Factor 2 (KEAP1:NRF2) Protein–Protein Interaction with High Cell Potency Identified by Fragment-Based Discovery. Davies, T.G., Wixted, W.E., Coyle, J.E., Griffiths-Jones, C., Hearn, K., McMenamin, R., Norton, D., Rich, S.J., Richardson, C., Saxty et al. *J. Med. Chem.* **2016**, *59*:3991-4006

14 Bricogne G., Blanc E., Brandl M., Flensburg C., Keller P., Paciorek W., Roversi P, Sharff A., Smart O.S., Vonrhein C., Womack T.O. **2017**. BUSTER version 2.11.7. Cambridge, United Kingdom: Global Phasing Ltd.

15 Emsley, P.; Lohkamp, B.; Scott, W. G.; Cowtan, K., Features and development of Coot. Section Title: *Biochemical Methods* **2010**, *66*, 486-501.

16 Smart, O. S., Womack, T. O., Sharff, A., Flensburg, C., Keller, P., Paciorek, W., Vonrhein, C. and Bricogne, G. **2011**. Grade, version 1.2.20. Cambridge, United Kingdom, Global Phasing Ltd. https://www.globalphasing.com.

Details from the data processing and refinement are reported in table S1. Pictures were generated with the software PyMol[17].

Table S3 - Statistics from crystallographic data reduction and refinement. Values in parenthesis refers to the highest resolution shell

Compound	**P3-F**	**P8-F**
PDB accession code	8Q1Q	8Q1R
x-ray source/synchrotron	BioMAX - MAX IV	BioMAX - MAX IV
Space group:	$P6_1$	$P6_1$
Cell constants: a b c (Å)	103.4 103.4 55.7	103.0 103.0 55.7
Resolution range (Å)	89.6 – 1.38	89.2 – 1.35
Highest resolution shell (Å)	1.47 - 1.38	1.43 – 1.35
Ellipsoidal resolution limit (direction) (Å) 0.894 a* - 0.447 b* 1.34, b*, c*	1.38, 1.38, 1.74	1.34, 1.34, 1.70
Completeness, ellipsoidal (%)	95.3 (69.4)	88.0 (52.5)
Completeness, spherical (%)	76.2 (22.7)	71.1 (20.9)
Total number of observations	537635	523215
Reflections, unique	53417	53141
Redundancy	10.1 (5.8)	9.8 (4.3)
R merge[1]	0.076 (1.19)	0.082 (0.77)
I/SigI[2]	14.9 (1.5)	12.3 (1.5)
CC1/2	0.999 (0.490)	0.998 (0.577)
R value $_{overall}$ (%)[3]	17.4	19.6
R value $_{free}$ (%)[4]	18.9	22.9
Bond lengths rmsd (Å)	0.008	0.012
Bond angles rmsd (º)	1.02	1.21
In most favoured regions[6] (%)	90.8	90.0
In additional allowed regions[6] (%)	8.3	9.6
In generously regions[5] (%)	0.0	0.0
In disallowed regions[5] (%)	0.8	0.4

[17] The PyMOL Molecular Graphics System, Version 1.7: Schrödinger, LLC, New York, NY, **2010**.

1 $R_{\mathrm{merge}} = \;_{hkl} [(\Sigma_i |I_i - \langle I \rangle|)/ \Sigma_i I_i]$

2 I/sigI avg is the mean I/sig for the unique reflections in the output file

3 $R_{\mathrm{value}} = \;_{hkl} ||F_{\mathrm{obs}}| - |F_{\mathrm{calc}}|| / \;_{hkl} |F_{\mathrm{obs}}|$

4 R_{free} is the cross-validation *R* factor computed for the test set of 5 % of unique reflections

5 Ramachandran statistics as defined by PROCHECK[18]Q1

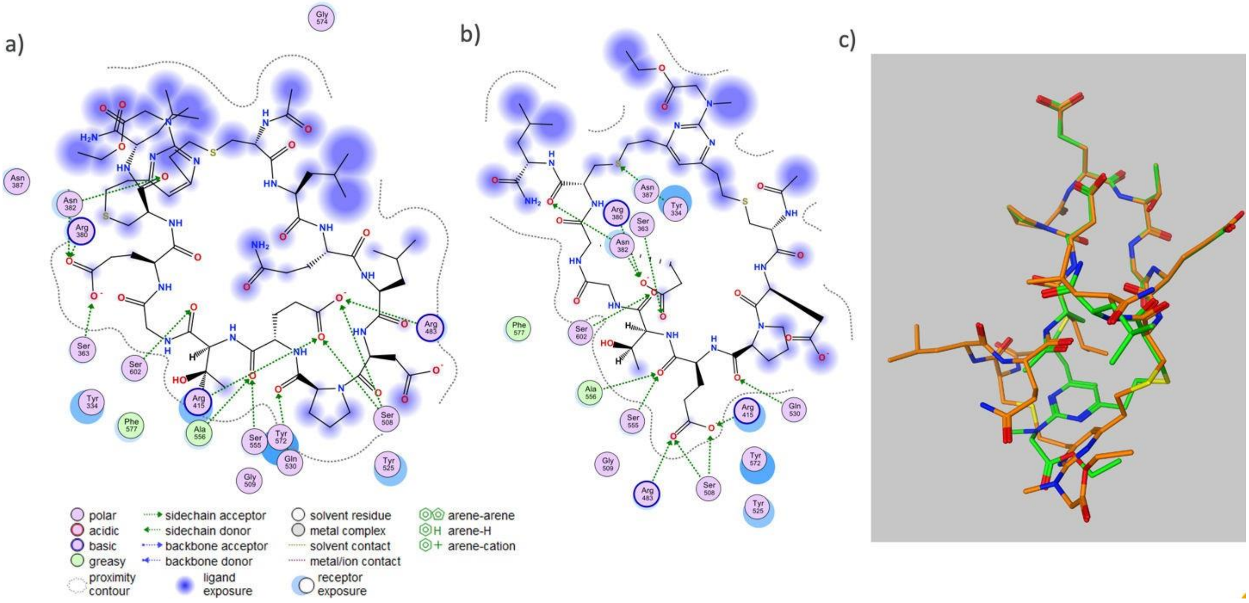

Figure ES_1 – a) Ligand interaction diagram of P3-F with the Keap1 Kelch domain (PDB: 8Q1Q); b) Ligand-interaction diagram of P8-F with the Keap1 Kelch domain (PDB: 8Q1R). Legend in the figure. c) Overlay of the two peptides P3-F in orange and P8-F in green) showing key residues maintain the same conformation whilst the solvent expose staples adopt a different conformation

[18] Laskowski, R.A., MacArthur, M.W., Moss, D.S. and Thornton, J.M. PROCHECK: a program to check the stereochemical quality of protein structures. *J. App. Cryst.* **1993**, *26*, 283-291.

Cellular Biology Experimental

BEAS-2B NQO1 Assay

The assay identifies compounds that upregulate NQO1 gene expression in BEAS-2B cells.

BEAS-2B cells were plated in BEGM (Lonza, Switzerland) medium without FBS into 384 Corning CellBIND plates (Corning, USA) at a density of 10,000 cells/well and incubated in the presence of the peptides for 24h. Cells were lysed in RLN buffer (140 mM NaCl, 1.5 mM $MgCl_2$ 50 mM Tris-HCl pH 8.0, 0.5% (v/v) Nonident P-40) supplemented with the RNAsecure reagent (Thermo Fisher Scientific, USA). The Cell-to-Ct Fast Advanced RT kit (Thermo Fisher Scientific, USA) was used to perform cDNA synthesis. All PCR reactions were carried out in MicroAmmp EnduraPlate optical 384-well clear plates (Thermo Fisher Scientific, USA). For the analysis of NQO1 expression, the cDNA was diluted 27 times in TaqMan Fast Advanced Mater Mix (Thermo Fisher Scientific, USA) supplemented with the NQO1 specific probe HS00168547_m1 (Thermo Fisher Scientific, USA) and the GAPDH specifc probe Hs99999905_m1 (Thermo Fisher Scientific, USA). The gene expression analysis was performed in 10µl final reaction volume using QuantStudio 7 Flex (Thermo Fisher Scientific, USA). Ct values were calculated using QunatStudio 7 software. Calculation of normalized relative expression levels and EC_{50} values from four parameter logistic fit was performed using Genedata screener software (Genedata, Switzerland). Additionally, two-point normalization using neutral and stimulator controls (neutral control: DMSO, 1 µM of a stimulator control) was applied.

CellTiter Glo Assay

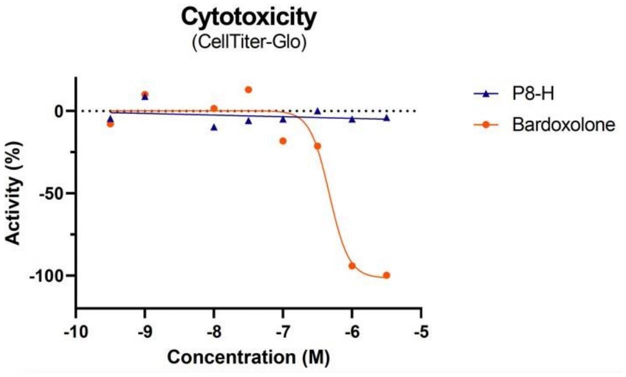

*Figure ES_2 - Celltiter Glo assay of lead peptide **P8-H** compared to the cytotoxic small molecule Bardoxolone. The assay was performed according to manufacturer instruction and using BEAS-2B cells*
